# Cerium Oxide Nanoparticles: Recent Advances in Tissue Engineering

**DOI:** 10.3390/ma13143072

**Published:** 2020-07-09

**Authors:** Motaharesadat Hosseini, Masoud Mozafari

**Affiliations:** 1 Department of Biomedical Engineering, Amirkabir University of Technology, Tehran 1591634311, Iran; motahare.s.h@aut.ac.ir; 2 Department of Tissue Engineering & Regenerative Medicine, Faculty of Advanced Technologies in Medicine, Iran University of Medical Sciences (IUMS), Tehran 1449614535, Iran

**Keywords:** cerium oxide, nanoceria, tissue engineering, physicochemical properties

## Abstract

Submicron biomaterials have recently been found with a wide range of applications for biomedical purposes, mostly due to a considerable decrement in size and an increment in surface area. There have been several attempts to use innovative nanoscale biomaterials for tissue repair and tissue regeneration. One of the most significant metal oxide nanoparticles (NPs), with numerous potential uses in future medicine, is engineered cerium oxide (CeO_2_) nanoparticles (CeONPs), also known as nanoceria. Although many advancements have been reported so far, nanotoxicological studies suggest that the nanomaterial’s characteristics lie behind its potential toxicity. Particularly, physicochemical properties can explain the positive and negative interactions between CeONPs and biosystems at molecular levels. This review represents recent advances of CeONPs in biomedical engineering, with a special focus on tissue engineering and regenerative medicine. In addition, a summary report of the toxicity evidence on CeONPs with a view toward their biomedical applications and physicochemical properties is presented. Considering the critical role of nanoengineering in the manipulation and optimization of CeONPs, it is expected that this class of nanoengineered biomaterials plays a promising role in the future of tissue engineering and regenerative medicine.

## 1. Introduction

Numerous examples have been found where technology plays a leading role in enhancing human life by providing human tissues and nanomedicine products. Over the years, many biomedical efforts have been made to restore functions lost owing to disease or trauma [1,2]. For example, if we consider cancer, marked advances began from the time of the first modern treatment, which was developed by the use of X-rays (probably at the end of the 1800 s) and continued to offer state-of-the-art therapeutic approaches throughout the last decade [3,4]. Cell-based therapies, particularly tissue engineering, are being investigated as a promising repair platform [5,6]. The physiological levels of intracellular reactive oxygen species (ROS) such as radicals do play several functional roles, like cell signaling, and these reactive species are typically released as by-products of oxygen metabolism [7]. With this in mind, ROS is one of the earliest signals that drives repair as well as regeneration. Recently, this beneficial capacity of oxidative stress in regeneration has garnered much attention [8]. In spite of this, environmental stressors such as UV, ionizing radiations, pollutants, and heavy metals, and xenobiotics such as antiblastic drugs, have been found to be involved in the notable elevation of ROS production. These observations are considered a threat to the balance in the body that results in cell and tissue impairment (detrimental oxidative stress) [7]. In normal cells, the presence of deregulated oxidative stress triggers death pathways [9].

Additionally, inflammatory responses or graft rejections by the host constitute some of the most formidable challenges for all kinds of implanted biomaterials [10,11,12,13]. Indeed, inflammatory cells secrete many reactive species at the site of inflammation which, consequently, culminates in worsened oxidative stress [14]. On the other hand, a variety of reactive species can stimulate intracellular signaling cascade that has promotive effects on proinflammatory gene expression [15,16]. Therefore, inflammation and oxidative stress are closely connected to pathophysiological events and associated with a wide range of chronic diseases, such as diabetes [17], hypertension and cardiovascular diseases [18], neurodegenerative diseases [19], alcoholic liver disease [20], chronic kidney disease [21], cancer [22], and aging [23]. In tissue engineering, numerous strategies have been proposed to tackle these issues [24,25,26,27]. For example, it has been reported that biocompatible materials with sustainable scavenging abilities are effective for protecting de novo tissue from inflammation [28].

Cerium oxide nanoparticles (CeONPs; nanoceria) have the potential to exert an anti-inflammatory effect for engineered tissues due to its in vitro and in vivo capability of scavenging reactive species, suppressing inflammation, mitigating cytokine levels, and providing cell protection [29,30,31,32]. There have been many pieces of evidence in favor of the CeONP’s protective role for several mammalian cell types, such as neural [33,34], retinal [35], hepatic [36], cardiac [37], breast [38], and cartilage cells [28], from oxidative stresses and inflammatory responses [39]. Intriguingly, CeONPs reduce cancer cell viability and invasion, while showing nontoxicity to normal cells [40,41,42,43]. CeONPs have carried harmful impacts on human broncho-alveolar cancer cells via the production of free radicals and membrane damage that are associated with decreased cell viability [44]. Moreover, scientists have successfully linked folic acid to CeONPs, which helped increase the uptake of coated CeONPs in ovarian cancer cells and induce cell death by generating ROS [45] In the pertinent literature, the pro-oxidant, cytotoxic effects of cerium oxide (CeO_2_) nanoparticles (CeONPs) are also highlighted under cellular and animal experiments [46,47,48,49,50]. These examples emphasize the potential prospective use of nanomaterials, CeONPs in particular, for the future.

The success of biomedical nanotechnologies is connected to the development of non-toxic restorative and therapeutic biomaterials [51,52,53]. The adverse effect of nanomaterials on humans has become the primary concern of the health sector, because NPs are capable of crossing biological barriers and gaining access to cells, while larger-sized particles typically fail. It is generally known that toxicity is inversely related to the NP’s size [54]. However, interactions at the nano–bio interface may violate this well-accepted relationship. Due to their unique physicochemical properties in a variety of biological systems, conflicting toxic outcomes have been reported in previous studies. Therefore, to deepen our current knowledge concerning the toxicity of NPs, further work is imperative, since the interaction between NPs and biosystems appears more complex than previously thought. This review will provide insights into how cerium oxide nanoparticles (CeONPs; nanoceria) have the potential to exert an anti-inflammatory effect on engineered tissues due to its in vitro and in vivo capabilities of scavenging reactive species, suppressing inflammation, mitigating cytokine levels, and providing cell protection [29,30,31,32]. There have been many pieces of evidence in favor of the CeONP’s protective role for several mammalian cell types, such as neural [33,34], retinal [35], hepatic [36], cardiac [37], breast [38], and cartilage cells [28], from oxidative stresses and inflammatory responses [39]. Intriguingly, CeONPs reduce cancer cell viability and invasion while showing nontoxicity to normal cells [40,41,42,43]. CeONPs have carried harmful impacts on human broncho-alveolar cancer cells via the production of free radicals and membrane damage, which are associated with decreased cell viability [44]. Moreover, scientists have successfully linked folic acid to CeONPs, which helped increase the uptake of coated CeONPs in ovarian cancer cells and induced cell death by generating ROS into recent biomedical enhancement or regeneration in certain tissues, and, within several kinds of NPs, it will concentrate on CeONPs. Moreover, this review deciphers the association between CeONPs’ toxicity and their physicochemical properties for applications in tissue engineering.

## 2. Tissue Engineering Applications of Nanomaterials—New Roles for an Old Player

Tissue engineering is intended to create constructs from cells and scaffolds in an attempt to restore or repair lost tissues and organs and avoid lengthy, complex, and rarely available organ transplants. The nano-sized design of a tissue-engineered implant renders biocompatibility, establishes a precise resemblance to the native extracellular matrix, builds a physiologically relevant biomechanical niche, and gives access to biological factors that are essential for functional tissue regeneration [55,56]. Current progress in nanotechnology facilitates the synthesis or fabrication of biocompatible nanomaterials including NPs, nanoporous scaffolds, nanopatterned surfaces, nanofibers, nanowires, and carbon nanotubes [57,58]. These categories of nanomaterials are found with specialized applications in regenerative medicine and tissue engineering. For example, NPs are mainly utilized as carriers for the targeted and controlled release of growth factors, antioxidants, and anti-inflammatory drugs. Besides this, they have the capability of incorporating into scaffolds in order to regulate mechanical features, hardness, biodegradation, and many others [59,60]. Nanoporous materials, developed by applying sol-gel methods, etching techniques, and electrochemical processes, show an augmented surface area, pore-size related diffusion activities, excellent protein adsorption, and cell integration. These effects make them a good candidate for tissue engineering, particularly bone tissue engineering [61,62]. Nanopatterned surfaces consist of structures like pillars, ridges, and other topographical features that enable the tuning of mechanical properties and surface area. In this very specific category, surfaces play a pivotal role in obtaining extensive cellular responses such as stem cell differentiation and the prevention of fibrotic responses [63,64,65]. Nanofibrous biomaterials, fabricated by electrospinning techniques, for the most part, are useful for rebuilding the architecture of the extracellular matrix with therapeutic benefits [66,67]. Carbon nanotubes can afford to reinforce bioengineered scaffolds for stiffness and add sophisticated properties, namely electrical conductivity and controlled drug delivery. These unique activities support the use of carbon nanotubes in cardiac and neural tissue engineering [68,69]. Primary applications of nanomaterials deal with the loading and release of deliverable factors that can serve as exogenous cues to activate the molecules involved in tissue engineering. These conventional nanomaterial-based approaches face some challenges in terms of activity preservation, sustained release, and preparation cost [70]. As an innovative alternative, CeONPs, with their innate redox-cycling ability, open up perspectives in active tissue regeneration.

## 3. Cerium Oxide Nanoparticles and Molecular Targets in Redox Regulation

Lanthanide-derived NPs have been used in nonmedical industries to a great extent. Such successful utility may provide the basis for biomedical applications, but the complex nature of physiological systems acts as a hurdle for effective CeONP-based therapy. One health concern appears to be the genotoxicity [71] and immunotoxicity [72] of these engineered CeONPs. Before considering the toxicity of CeONPs and the effect of toxicity on tissue engineering, we have first discussed their various emerging applications in this field. Recent studies have identified a wide range of antioxidant, antibacterial, anti-inflammatory, and antiapoptosis activities for CeONPs [30,73,74] that make these NPs suitable for a wide range of applications in advanced tissue engineering. Contrary to the initial thought that oxygen vacancies along with redox-cycling between cerium in 3+ and 4+ states are responsible for the antioxidant properties of CeONPs [75,76], now, researchers have presented the finding that redox-cycling exclusively lies behind all the antioxidant activities [77]. As a result, the surface ratio of Ce^3+^ to Ce^4+^ accounts for all of the CeONP’s biological functions, particularly tissue regeneration.

**Figure 1:**
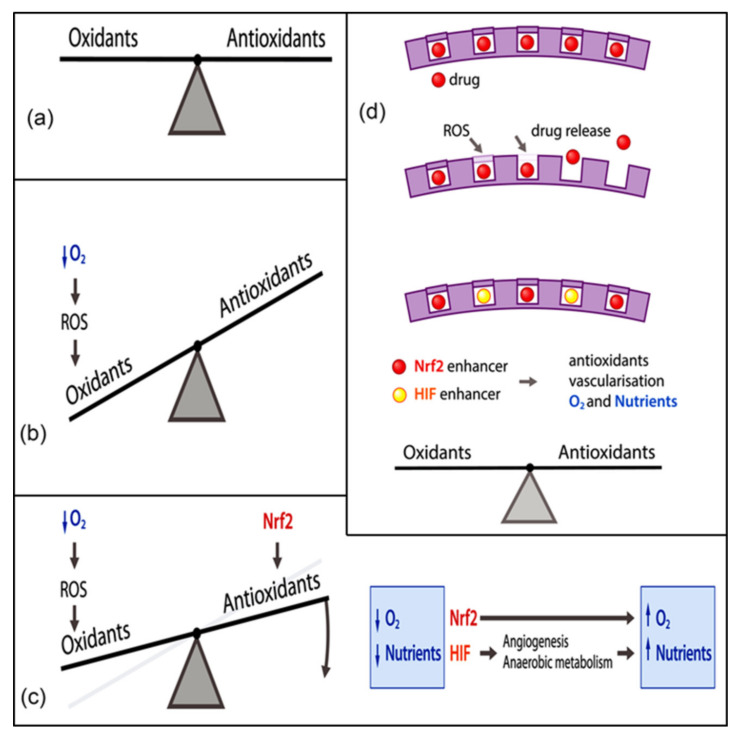
(**a**) Under normal physiological conditions, the cells in our body have tight and stable control over the dynamic redox balance that reflects the equilibrium between ROS and antioxidants. The cell encounters a diverse range of oxidant and antioxidant challenges in a continuous manner and responds to them with the presence of endogenous ROS generators and the modulation of endogenous antioxidant activities. (**b**) If the amount of oxidants outweighs that generated by the endogenous antioxidant systems, the balance will be interrupted and result in a phenomenon known as oxidative stress. This can be exemplified by hypoxia, caused by the exposure of cells to a lower amount of oxygen. (**c**) The redox imbalance can be treated by using the endogenous modulators of the endogenous antioxidant system. As an example, the increment of the transcription factor Nrf2 is connected to the enhanced levels of endogenous antioxidant systems [92]. Additionally, elevating the transcription factor HIF (hypoxia inducing factor) gives rise to anaerobic metabolism, as well as angiogenesis, ending up with glutaminase-mediated GSH (glutathione) production, thereby boosting endogenous antioxidant systems [93,94]. (**d**) A more elaborate strategy for restoring the redox imbalance is to develop a biomaterial with the ability to carry and release drug molecules upon additional ROS or oxidative stress. Reprinted with permission from [26]. Whiley reports open access.

In body tissues and organs, there is a tightly controlled balance between oxidants and antioxidants to retain their functions (Figure 1a). Oxidants refer to the compounds that have ROS generation capacities, while, on the contrary, antioxidants can afford to scavenge these radical species and retard the oxidation processes of other compounds [78]. Oxidants and antioxidants trigger the reactions that, as a whole, are considered redox reactions or are respectively called reduction and oxidation reactions [79]. Cells are regularly exposed to large amounts of oxidants because of endogenous cues such as increased aerobic metabolism or exogenous cues such as ionizing radiation [80,81] (Figure 1b). On the other side of the coin, each and every cell benefits from a number of endogenous antioxidant systems, such as the glutathione (GSH) system, thioredoxin system, various vitamins, and protective enzymes (e.g., catalase (CAT) or superoxide dismutase (SOD)) that are sensitive to the redox balance and are able to recover the redox balance whenever required. In this scenario, several redox-regulated transcription factors take part, notably nuclear factor erythroid 2-related factor 2 (Nrf2) or hypoxia inducing factor (HIF) (Figure 1c). The endogenous antioxidant defense mechanisms are compartmentalized at conserved subcellular sites. For instance, mitochondria, in which aerobic metabolism occurs, are rich in GSH and SOD, whereas vitamin E can be detected in the plasma membrane for the most part [82]. In the presence of oxidative stress, the generation of oxidants like ROS augments to excess, as compared to that of endogenous antioxidants, in such a way that cells fail to maintain the balance [83]. Despite the evidence that a short-term and relatively small upregulation of ROS is of the utmost importance for the redox signaling that plays a pivotal role in multiple processes, including inflammation [84,85] or angiogenesis [86,87,88], a long-term and relatively large elevation of ROS leads to the impairment of major cellular macromolecules, deoxyribonucleic acid (DNA), proteins, or lipids that is more likely to induce the formation of many pathological conditions, such as diabetes [89,90] and neurodegenerative diseases [91].

Therapeutic approaches targeting the activation of Nrf2 and HIF, as redox-regulated transcription factors, have been associated with the future direction for clinical practice (Figure 1d). The wide variety of CeONP activities, on the other hand, is ascribed to its thermodynamic efficiency of redox-cycling between 3+ and 4+ states on its surface [76] and its notable characteristic of absorbing and releasing oxygen [95]. Therefore, the engineered biomaterials of these views constitute an essential part of research studies addressing the potential use of CeONPs in tissue engineering. Currently, Passi et al., for example, designed a multifunctional silk fibroin-based carrier for integrating the delivery of antioxidant and imaging agents [96]. Accordingly, silk fibroin NPs containing sulforaphane (antioxidant drug) (SFSNPs) were fabricated by means of a one-step desolvation method. Then, on the surface of these anionic NPs, cationic CeONPs were coupled with polyethylenimine (PEI) passivated carbon dots (CDs) by electrostatic interactions in order to produce self-assembled CeONP-CD@SFSNP nanocomposites. Moreover, CDs were created from mulberry leaves (*Morus indica*), as a green source of carbon, and bPEI, as a passivating agent, in an attempt to develop positively charged CDs. The resulting CDs worked as molecular probes via the emission of green fluorescence, while CeONPs were added to raise the antioxidant potential because of their special redox features. The entrapment efficiency of sulforaphane was 65.21%, and the average hydrodynamic size of the NPs was 365 nm. The as-prepared CeONP-CD@SFSNP nanocomposites effectively reduced ROS levels by simultaneously enabling the imaging of the lung cancer cells in H_2_O_2_ induced oxidative stress. In this case, the Nrf2 pathway was triggered by sulforaphane, and the antioxidant activity was promoted by the SOD and CAT mimetic activity of CeONPs, owing to mixed valency. Besides this, CDs augmented antioxidant activity. The whole nanozyme is potentially suitable for different pulmonary disorders, such as chronic obstructive pulmonary disease [96]. Despite the fact that the CeONP-CD@SFSNP nanocomposites were initially developed for cancer therapy, their ability to scavenge free radicals offers additional regenerative applications. Actually, oxidative stress can hamper cellular growth and proliferation in tissue engineering. It is worth noting that redox regulation is mediated by the activation of Nrf2 as the molecular target. As a result, these nanocomposites can be a good example of an innovative strategy to restore the oxidant and antioxidant balance necessary in tissue regeneration by collectively suppressing the oxidative insults and stimulating the Nrf2 pathway.

As for the second molecular target, which is the HIF pathway, Nethi et al., aiming to augment the pro-angiogenic potential of CeONPs, employed functionalization strategies [97]. In this regard, they conjugated aqueous, dispersible CeO_2_ and trivalent metal (Sm) ion-doped CeO_2_ (SmCeO_2_) NPs with hydrophilic biocompatible and antifouling (6-(2-[2-[2-methoxy-ethoxy]-ethoxy]-ethoxy) -hexyl)triethoxysilane moieties. These nanoconjugates were observed with reduced or optimal ROS levels in treated endothelial cells. As a result, the functional nanoconjugates of SmCeO_2_ stimulated the proliferation of endothelial cells and brought about the growth of blood vessels in a chick embryo. Moreover, the nanoconjugates promoted the expression of pro-angiogenic markers, including HIF-1α [97]. The synergism between the inhibition of cellular ROS production and the activation of the HIF signaling pathway provides strong pro-angiogenic properties and holds promise for wound healing and cardiac tissue engineering.

## 4. Recent Advances in Tissue Engineering

In recent years, medical care has undergone a positive shift from the conventional concept of organ replacement to the emerging approach of tissue regeneration by introducing the cutting-edge technologies of tissue engineering as well as regenerative medicine. Biomaterials have entered the medical care system and brought about marked breakthroughs for a long period. Metals tend to be used as the major parts of effective initial biomaterials and remain an attractive area for the design of new regenerative therapies, which are subsequently being substituted by natural tissues or their derivatives [98,99]. In biosystems, CeONPs are capable of carrying both pro-oxidative and antioxidant effects [100,101,102]. In other words, these NPs can serve as pro-oxidants by generating ROS involved in cell damage and consequently cell death and via altering the intracellular redox status [102]. On the contrary, CeONPs are considered direct antioxidants given their free radical scavenging capacity, thereby preventing cell death in oxidative stress [100,101,103]. Therefore, CeONPs have been found to possess significant capabilities in tissue repair and regenerative medicine. In the following, more explanations are presented about the role of CeONPs based on their pro-oxidative and antioxidant properties.

### 4.1. Stem Cell Differentiation

It is well-known that the repair of tissue injury by biomaterials can include two chief processes: constructive remodeling (i.e., the replacement of lost tissue by parenchymal cells of the same type) and the formation of a fibrous capsule (i.e., replacement by connective tissue). These two processes are typically regulated by either the proliferative ability of the cells in the target tissue which receives the scaffold and the extent of the injury which contributes to the destruction or the persistence of the tissue extracellular matrix at the site of implantation [104]. One of the key factors determining the growth capacity of cells in tissue engineering is the development of scaffolds that are capable of mimicking the adhesive signals for the proliferation of parenchymal cells and the generation of a native extracellular matrix [105,106]. In the context of tissue engineering, stem cells are popular as the building blocks of regenerative medicine-based strategies. Scaffolds improve constructive remodeling on the basis of the mechanisms that involve stem/progenitor cells [107]. Thus, two different categories of studies have been carried out on microenvironmental conditions and the interactions of cells with CeONPs (Table 1).

The first category of investigations indicates that CeONPs as additives to scaffolds fabricated for tissue engineering can mimic the natural cell surrounding in vivo (niche) and affect the behavior of stem cells, such as their migration, proliferation, and differentiation. An initial investigation has reported that CeONPs can augment the proliferation of human mesenchymal stem cells (hMSCs) by counteracting oxidative stress, which occurs in normal metabolism. Furthermore, CeONPs facilitate their differentiation, as characterized by a considerable production of collagen [117]. This evidence provides the basis for further studies on stem cells in cardiac tissue engineering. For example, Mandoli et al. fabricated CeONPs/poly lactic-co-glycolic acid (PLGA) films and evaluated the capacity of cardiac stem cells (CSCs) and MSCs for cardiac tissue engineering [118]. An increased cell density was observed for PLGA loaded with 5 wt.% and 10 wt.% CeONPs as opposed to unloaded films. This growth is the result of the CeONP’s scavenging activity, which is sensitive to the high loading of the CeONPs (20 wt.%) since it causes the pronounced agglomeration of the CeONPs and damps its free radical scavenging activity. Additionally, the presence of the CeONPs led to cell alignment. The CSCs responded to the loaded films in terms of viability, proliferation, and spatial growth, which can be explained by the roughness, stiffness, and surface micro-topography of the PLGA scaffold or the chemical nature of the CeONPs. It is worth noting that the incorporation of the CeONPs produced physical and morphological changes in the roughness and stiffness of the PLGA composites. In order to better understand the role of the CeONPs, another PLGA composite was developed by 6 wt.% TiO_2_ with a relatively similar roughness, particle size (5–8 nm), and stiffness. Cell studies revealed that greater attachment and cell density were achieved for CSCs cultured on the CeONP-loaded scaffolds than the TiO_2_-loaded composites. The change in cell type from CSCs to MSCs culminated in better cell proliferation for the CeONPs than TiO_2_. Even the random distribution of the CeONPs in the PLGA matrix was associated with a preferred growth and proliferation. These findings can be justified by the chemical nature of the CeONPs that have stronger biochemical interactions with cells as compared to TiO_2_. In other words, Ti may be found in both Ti^4+^ and Ti^3+^ valence states. However, unlike the CeONPs, a trivalent state shows more stability and 3+ ions are fewer in number. Therefore, TiO_2_ can react with ROS, but it is less effective than the CeONPs, which may account for the weak biochemical interactions between TiO_2_ and cells. In light of these results, a chemical cue, probably related to the CeONPs’ antioxidative properties that are not affected by loading in the scaffold, is responsible for the enhanced cell behavior [118].

With the same thought that free radicals can interfere with cellular growth and CeONPs act as potent antioxidant agents, Karakoti developed a hypothesis that CeONPs can play the role of an oxygen molecule scavenger and improve bone tissue engineering [119]. Given that hMSCs are quick to detect and respond to toxic compounds, such as hydroxyl radicals or hydrogen peroxide, they proposed to the addition of CeONPs to the host matrix (three-dimensional bioactive glass foam). For this application of CeONPs, they controlled the size of NPs to within 3–5 nm to minimize the interfering effect of CeONPs on the matrix. Moreover, the CeONPs were synthesized in water and dextran, with the former exhibiting better radical scavenging properties. Cell culture studies in the absence of osteogenic factors showed a high level of ALP (alkaline phosphatase) expression, indicative of the enhanced osteoblastic differentiation of hMSCs in the CeONP-containing matrix. The ALP enzymatic activity was greater in the CeONPs synthesized in water. Interestingly, the CeONPs prepared in water were found to possess higher amounts of collagen, meaning an increased deposition of the extracellular matrix [119]. The antioxidant behavior of CeONPs in the biological world may be linked to oxygen vacancies. Theoretically, superoxide radicals are instantly transformed into peroxide on the surface of the reduced CeONPs [120]. The attachment of the peroxide molecules to the surface is stronger than that of the oxygen molecules. Accordingly, CeONPs have the ability to regulate the molecular oxygens available to cells due to their oxygen buffering effects that make the release and absorption of oxygen easy. It is widely accepted that the oxygen level is critical for cellular growth and proliferation. Thus, the enhancement of osteoblastic differentiation as a result of the CeONP’s incorporation may be attributed to better control of the oxygen level.

Additionally, scaffolds are of the utmost importance during the formation of a fibrous capsule, which hinders the contact between cells and the biomaterial and impairs the process of constructive remodeling. These events arise from the response of the immune system, where macrophages are a significant part [121]. Moreover, they participate in the modulation of inflammation, which can be initiated by the overproduction of free radicals [122,123]. Li et al. propose the new scenario of macrophage-mediated inflammatory responses for bone healing, since an active type of macrophages (M2) secretes growth factors to ameliorate the migration and osteogenic differentiation of MSCs [124]. They exploit the addition of CeONPs to hydroxyapatite (HA) coatings for controlling the inflammatory response of macrophages and promoting the osteogenic activity of bone marrow-derived mesenchymal stem cells (BMMSCs). To this end, the HA coating was doped with 10 wt.% and 30 wt.% of the CeONPs, leading to a Ce^3+^/(Ce^3+^ + Ce^4+^) ratio of 29.49% and 33.79%, respectively. Biological evaluations showed the adhesion of BMMSCs with similar spread shapes on HA coatings that were both modified with the CeONPs and not. The cell proliferation, ALP activity, and mineralization were significantly upregulated with an increase in the CeONP content. Furthermore, the expression levels of osteogenic genes like Runx2, ALP, and OCN were augmented considerably for the BMMSCs cultured on the HA coating with the highest amount of CeONPs (30 wt.%). ALP is considered an early marker of the osteoblast lineage, with OCN being a marker of the mature osteoblastic phenotype and pointing out calcium deposition. Therefore, the CeONPs could stimulate the calcium channel on the surface of the BMMSCs to improve their osteogenesis [70]. The much greater mRNA levels of BMP2, BMPR1, BMPR2, Smad1, Smad5, and Smad8 on the HA coating doped with 30 wt.% CeONPs exhibited the involvement of the Smad-dependent BMP (bone morphogenetic protein) signaling pathways in the osteogenic differentiation of BMMSCs. These pathways could support the expression of bone structural proteins including ALP and OCN [125]. Further analyses indicated a content-dependent downregulation of certain surface markers (CCR7 and CD11c), as well as proinflammatory cytokines (Interleukin (IL) 6 and tumor necrosis factor α), meaning that the modified HA coating did not trigger the polarization of macrophages toward the M1 phenotype. It can prevent interactions between the bone tissue and the biomaterial. By contrast, a tendency for the polarization toward the M2 phenotype was observed for the HA coating loaded with 30 wt.%, as characterized by the upregulation of surface markers CD206 and CD163, along with anti-inflammatory cytokines IL-10 and IL-1ra. Besides this, enhanced expression levels of osteoblastogenesis-related genes BMP2 and transforming growth factor β1 (TGF-β1) occurred for the 30 wt.% incorporated HA coating. These two genes can promote the migration and recruitment of endogenous stem cells [126]. Intriguingly, it significantly reduced the macrophage ROS content, which can explain the downregulation of the above-mentioned surface markers and proinflammatory genes [110].

The second category represents a new design of artificial niches in tissue engineering where the CeONPs are dispersed into the culture medium of stem cells. In this very specific application context, CeONPs are a powerful tool to control oxidative stress within the normal physiological levels. This approach can be used for the generation of a sufficient number of target cells for clinical administrations. For instance, adult progenitor cells, known as the precursors of all differentiated cells in a certain germ layer, exist in approximately each and every part of the body. Since these cells are able to self-renew and show commitment to a specific cell lineage, they are involved in the processes of tissue repair as well as physiological cell turnover. However, they live in distinct, very small regions of all body tissues with a critical niche [127], which necessitates more research on the growth of progenitor cells. Pagliari et al. utilized CeONPs to regulate the growth and proliferation of CPCs by hindering oxidative stress [116]. These cells did not express mature hematopoietic cell lineage markers (Lin^neg^) but were positive for the expression of stem cell antigen-1 marker (Sca-1^pos^), which correlated to multipotency and self-renewal in the bone marrow and heart. Ultrastructural analysis revealed the internalization of the CeONPs (5–8 nm) within CPCs as clusters of aggregated particles which were not entrapped by vesicular membranes. These observations suggest that the CeONPs did not trigger apparent cell structural damage and could be removed or inactivated. The exposure to the CeONPs did not affect the phenotype, growth, or differentiation of CPCs. They presented a well-organized cytoskeleton, proper focal adhesions, and a time-response effect. Moreover, they maintained their multipotency in adipogenic and osteogenic media, as evidenced by lipid vacuoles and calcium deposits, respectively. Upon the coculture with murine neonatal cardiac cells, the CeONP-treated CPCs were committed to cardiomyocytes and could acquire their typical markers by the 10^th^ day. The free radical scavenging properties of the CeONPs protected CPCs from oxidative stress by H_2_O_2_ in a time- and dose-dependent manner, while exposure to the CeONPs did not produce ROS significantly or induce functional modifications in these cells. What is more, the effective dose of the CeONPs might be determined by the amount of the uptaken CeONPs following 24-h exposure. The protection obtained by the cytosolic CeONPs lasted up to one week. These long-term properties include antioxidant actions, implying that an autoregenerative reactive cycle of cerium may be stimulated, thereby resulting in the persistent regeneration of the CeONP’s antioxidant activity. More likely, this effect can be attributed to the presence of a large Ce^3+^ or Ce^4+^ fraction. Interestingly, these findings highlighted a threshold which must be reached in the physiological interactions of the intracellular CeONPs before the emergence of the antioxidant effects. In addition, NPs with exact biological effects could internalize within cells and remain silent for a long time if biodegradation does not take place until their activation starts with intracellular and/or extracellular stimuli. However, all these events were reported in the absence of pH fluctuations and enzyme (e.g., SOD and CAT) actions, which should be evaluated in future studies [116].

**Figure 2:**
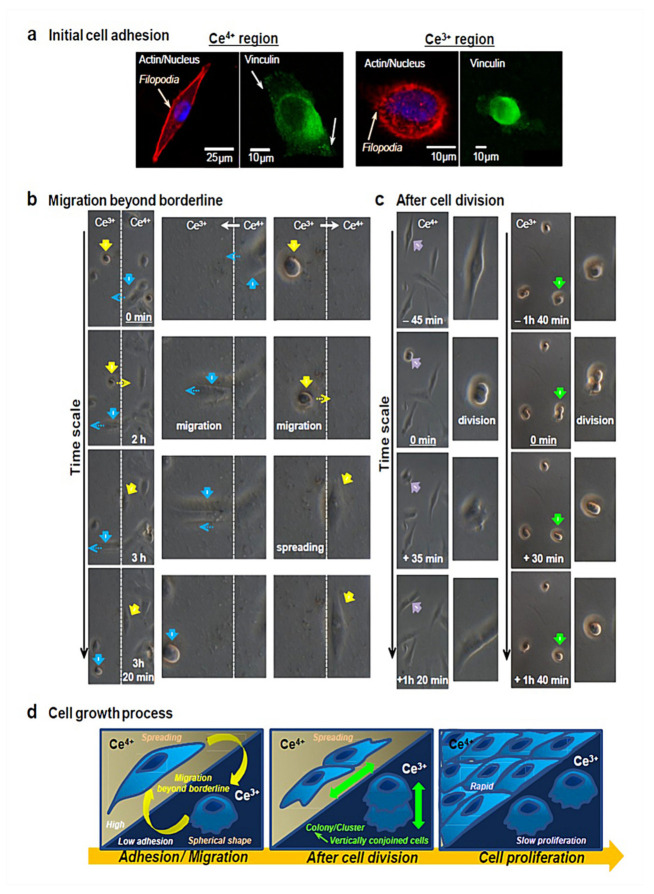
(**a**) As for the assessment of initial cell adhesion in Ce^4+^ and Ce^3+^ regions, actin filaments (red), nucleus (blue), and vinculin localization (green) are observed by applying immunofluorescence staining to cells by confocal microscopy. Cells adhere to Ce^4+^ regions (A-IV) and Ce^3+^ regions (B-III). The development of actin filaments and vinculin localization is evident in Ce^4+^ regions, whereas Ce^3+^ regions are found with no actin filaments and vinculin localization in the spherical-shaped cells. Interestingly, filopodia are active in both regions to a great extent. Filopodia are responsible for sensing the cell environment and building new adhesion contacts that further trigger cell migration and spreading. Therefore, cells are able to attach to Ce^3+^ sites by activating filopodia on a plasma membrane. (**b**) Cells can migrate beyond the borderline at a timescale of around 3h. Accordingly, cellular migration comes about from Ce^4+^ to Ce^3+^ regions (blue arrows) and vice versa (yellow arrows), as evidenced by reversible changes in the cellular morphology between spread shapes and spherical shapes. (**c**) In order to evaluate cell proliferation, cell morphology prior to/following cell division is observed. Cell division takes place in Ce^4+^ regions (purple arrows), with cells attaching to the substrate surface as per normal and then spreading. By contrast, cells undergo division on Ce^3+^ regions (green arrows), present as one cell, implying that cells are vertically conjoined and scarcely migrate on Ce^3+^ regions. When at least 8 h pass, cell separation and attachment are seen in Ce^3+^ regions, followed by initiated cell migration. Therefore, cells scarcely attach to Ce^3+^ regions, which, in turn, eases the development of a cell cluster/colony and supports strong cell–cell interactions but weak cell–material interactions. (**d**) All the events, namely cell adhesion, cell morphology, and proliferation, involved in cell growth in Ce^4+^ and Ce^3+^ regions are summarized schematically. Reprinted with permission from [109].

Taken together, the surface chemistry of CeONPs can be responsible for their antioxidant role and the stem cell’s behavior. As documented by Naganuma and Traversa, a higher amount of Ce^4+^ on the surfaces of CeONPs enables cell attachment, proliferation, spreading, and migration, whereas surfaces that are rich in Ce^3+^ inhibit these events. Such observations may be correlated with the greater hydrophobicity caused by Ce^3+^. The process of cellular growth, including initial adhesion (Figure 2a), morphology (Figure 2b), and proliferation (Figure 2c), is shown by confocal microscopy. Considering the whole cell growth process in the Ce^4+^ and Ce^3+^ regions, Ce valence states of 4+ and 3+ on substrates enable the promotion/inhibition of cell–material interactions, ending up with rapid/slow cell proliferation, respectively [109].

### 4.2. Angiogenesis Activity

The repair and regeneration of the injured tissues and organs fundamentally rely on the development of new vessels via angiogenesis, because it facilitates the access of the surrounding tissues, such as bones and nerves, to nutrition [128]. The lack of sufficient blood perfusion is a major problem that limits the clinical application of tissue engineering. The existing methods used for the improvement of blood vessel distribution deal with the delivery of angiogenic factors for the most part to enhance the proliferation, migration, differentiation, and vessel formation of endothelial cells and/or endothelial progenitor cells. This strategy is predominately associated with some limitations, as mentioned earlier. In emerging approaches involving the innate properties of CeONPs, marked angiogenic outcomes have been achieved in endothelial cells by combating the oxidative insults.

The primary effort in relation to the angiogenesis of CeONPs started with the study by Seal and his group, who dispersed these nanomaterials in the culture medium of human umbilical vein endothelial cells (HUVECs) [129]. Their biological studies indicated that CeONPs of either size (3–5, 10–15, 15–20, >25, and 50–60 nm) or shape (stars, polygonals, and nanorods (NRs)) did not reduce the proliferation of HUVECs, except for NRs. Another parameter in angiogenesis is tube formation. The exposure of chick embryos to CeONPs led to the notable, concentration-dependent induction of tube formation, which appeared to be a unique and intrinsic property of CeONPs. The CeONPs with an average size of <15 nm could only induce tube formation. In fact, an increase in the NP’s size can negatively influence the catalytic activity and therefore the absence of tube formation is expected. Nevertheless, no significant difference in tube formation was documented in response to the shape change. Interestingly, surface charge and Ce^3+^/Ce^4+^ ratio had no effect on the induction of tube formation. Concerning the effect of the culture medium on the CeONP’s surface charge, a shift to negative occurred that was not influential on tube induction. The synthesis method also played a role. The CeONPs prepared by the NH_4_OH precipitation method resulted in a weaker induction of endothelial tube formation and less robust vascular sprouting relative to those synthesized by the wet chemical method. As for the mechanism of action, further experiments revealed that the treatment of HUVECs with CeONPs produced pro-angiogenesis through intracellular vascular endothelial growth factor (VEGF) expression in a time- and concentration-dependent manner. Importantly, CeONPs did not induce angiogenesis via the activation of higher levels of ROS. The in vitro evidence showed that CeONP-induced angiogenesis was related to the tissue local oxygen concentrations and was indirectly handled by HIF-1α, which increased in the cytoplasmic and nuclear fraction. This mechanism could explain the effect of the synthesis method on tube formation in such a way that the wet chemical method generated 57% Ce^3+^ and a highly oxygen deficient surface, which can be more reactive in catalytic reactions and exhibit more angiogenic properties [129]. Therefore, CeONPs direct oxidative stress toward vessel formation via the regulation of oxygen concentration and the activation of HIF signaling.

Further studies in this regard have been continued for both soft and hard tissues. Almost all biological processes in the body’s tissues rely on the modulation as well as the transport of molecular oxygens. Given the oxygen-buffering capacity of CeONPs, the understanding of how they may impact angiogenic processes is of the utmost importance. In the following, more detail about the angiogenic effects of CeONPs is provided with respect to neovascularization in soft and hard tissue engineering. The previous research team in collaboration with Mattson and his colleagues reported the first example for cutaneous wound healing [130]. Adopting the same approach, CeONPs within a size range of 3–5 nm were dispersed in the culture medium of vascular endothelial cells. The cellular evaluation presented a significant increase in the rates of growth and migration. The vascular endothelial cells treated with CeONPs that were grown in a three-dimensional Matrigel matrix could form tubes considerably. This observation was confirmed by a significantly fast rate of wound closure in a murine model of skin wound healing. In skin tissue sections, the number of blood vessels was remarkably greater after the administration of CeONPs than no treatment in mice. These findings were associated with significantly low levels of 4-hydroxynoneanal (a lipid peroxidation product) protein adducts and nitrotryosine as the markers of oxidative stress in CeONP-treated mice. Thus, CeONPs accelerated neovascularization and reduced oxidative stress in wounded regions [130].

The initial thought that an increased ratio of Ce^3+^ to Ce^4+^ could induce neovascularization via the antioxidant activities of CeONPs [129] has been converted into a new insight into the redox-cycling between Ce^3+^ and Ce^4+^ that can be regulated by suitable dopant impurities. In other words, CeONPs can show catalytic activities without the need of Ce^3+^ sites [131,132]. The work by Nethi et al. contributed to this new insight and presented the notion that even a low ratio of Ce^3+^ to Ce^4+^ can make CeONPs angiogenic [97]. To this end, the role of dopant ions was highlighted. In this study, CeONPs and Sm-doped CeONPs were functionalized by organosilane (MTS-CeO_2_ and MTS-SmCeO_2_) to develop new blood vessels in vitro and in vivo. In this design, organosilane moieties were responsible for coordinating to the inner sphere of the surface cations by a strong ionic interaction. Both samples enhanced the viability and proliferation of endothelial cells (HUVECs and ECV-304 cells), with the doped NPs (MTS-SmCeO_2_) showing the better cellular behaviors. Therefore, the redox-altered MTS-SmCeO_2_ induced viability and proliferation efficiently. This observation implies that the doping of Sm ions carries an effect on the oxygen vacancy to render a differential Ce^3+^/Ce^4+^ redox state. In addition, these nanoconjugates caused little to no toxicity toward endothelial cells. The in vivo results demonstrated the promotion of new vasculature development. The antioxidant properties of the functionalized NPs were the possible pro-angiogenic signaling stimuli. Surprisingly, MTS-SmCeO_2_ held the optimal ROS level [97]. In the pertinent literature, it has been shown that HIF-1α is responsible for controlling the expression of genes that take part in angiogenesis. The stability of this transcription factor in the presence of intracellular oxygen contributes to the promotion of angiogenesis [133]. The functionalized CeONPs improved the expression of this angiogenic marker, along with p38 MAPK. Besides this, MTS-CeO_2_ and MTS-SmCeO_2_ contributed to the higher angiogenic properties than other samples (SmCeO_2_ and CeO_2_), which might arise from their greater aqueous dispersibility [97].

More recently, Park et al. have provided a deeper understanding of CeONP-induced revascularization irrespective of oxygen concentrations [134]. In this regard, they investigated the effectiveness of CeONPs in regenerating tissues during normoxia and a specific type of ROS-associated damage called critical limb ischemia. Their results showed that the synthesized CeONPs had an average size of 19.5 nm and a cubic morphology. The X-ray analysis revealed ROS consumption from Ce^4+^ to Ce^3+^ for the most part. An animal model of hindlimb ischemia in immunodeficient mice demonstrated no limb loss or necrosis after CeONP treatment in a dose-dependent fashion and significantly increased blood reperfusion depending on time. In addition, the animal studies exhibited that CeONPs could promote the formation of new blood vessels in immunocompetent mice. Comparatively, much more considerable recovery was found in immunodeficient mice, which could be justified by inflammatory reactions in immunocompetent mice that could interfere with the pro-angiogenic effects. The endogenous secretion of basic fibroblast growth factor (bFGF; which is involved in the fibroblast migration, proliferation, and deposition of the extracellular matrix throughout neovascularization), VEGF (which is the main promoter of angiogenesis), and hepatocyte growth factor (HGF; which mostly controls the inhibition of endothelial cell apoptosis and maintains homeostasis) was observed at high levels after CeONP treatment. The overall content of growth factors was correlated to CeONP doses. These angiogenic factors were stimulated without any exogenous angiogenic factors and linked to greater levels of reduction-oxidation factor 1-apurinic/apyrimidinic endonuclease (Ref-1/APE1), HIF-1α, and vascular endothelial growth factor A (VEGFA). The administration of CeONPs culminated in the regeneration of capillary structures and the maturation of blood vessels in mice. Therefore, the pro-angiogenic effects of CeONPs were mediated by Ref-1/APE1 signaling in addition to the HIF-1α pathway. The CeONP-induced angiogenesis, characterized by increased viability and tube formation in HUVECs, was related to the elevated expression of HIF-1α. This process occurred in normoxic conditions due to the high expression levels of endothelial nitric oxide synthase. A similar mechanism was observed in an ROS-excessive environment. Indeed, CeONP in response to great levels of oxidative stress preserved endothelial survivability through intracellular ROS scavenging that in the long run supported the formation of tubular networks and other endothelial cell functions. Therefore, the Ref-1/APE1 signaling pathway connected to the activation of HIF-1α can directly support the angiogenic effects of CeONPs [134].

There are some examples in favor of the angiogenesis of CeONP embedded in the engineered scaffolds. Xiang et al. modified cancellous bone at the surface level using CeONP and poly-_L_-lactic acid (termed as scaffold@CeONP) [70]. After this, they used the coculture of endothelial progenitor cells and MSCs on scaffold@CeONP and showed the improvement of cell viability and the differentiation process for endothelial progenitor cells. Such observations were related to the presence of CeONPs, rather than growth factor secretion by MSCs, which could promote the growth and differentiation of endothelial progenitor cells. This is because the cells were seeded in a non-contacting manner. The subsequent testing exhibited that the elevated expression level of VEGF from MSCs activated by CeONPs lay behind the enhanced growth, maintenance, and differentiation of endothelial progenitor cells. The mechanism whereby CeONPs increased VEGF expression was their contribution to the activation of the calcium channel at the MSC’s surface and the discharge of the calcium pool, which, in turn, raised the stability of HIF-1α. The murine studies demonstrated a higher level of vascularization for scaffold@CeONP than the scaffold itself, meaning that this bone construct could develop more blood vessels by stimulating the paracrine of angiogenic factors from MSCs. Moreover, the augmented penetration of blood vessels facilitated the formation of new bone tissues inside scaffold@CeONP. Therefore, intracellular free calcium might be involved in HIF signaling [70].

In another study, CeONPs were incorporated into electrospun polycaprolactone (PCL) scaffold [135]. The primary investigation revealed that this scaffold carried no effect on the normal physiology and function of blood or blood cells upon contact. Additionally, HUVECs displayed an obvious difference in cell viability after exposure to this nanocomposite scaffold. No membrane damage was observed in HUVECs cultured on the scaffold, whereas higher cell adhesion and cell numbers were evident relative to bare PCL. Intriguingly, higher numbers of capillary branches and newly formed blood vessels were found with this nanocomposite. In vivo studies in rats were indicative of a slight but marked increase in inflammatory responses but high cell proliferation and blood formation. Such angiogenesis was activated by HIF-1α, as shown by the upregulation of VEGF expression in the nanocomposite scaffolds. More importantly, the study presented a relationship between HIF-1α and the induction of inflammatory responses based on considerable levels of tumor necrosis factor α and cyclooxygenase genes when higher concentrations of CeONPs were embedded in the scaffolds [135].

Concerning nerve tissue engineering and axonal regeneration, Qian et al. proposed the use of the asymmetrical layer-by-layer three-dimensional manufacture technique to fabricate a collagen/CeONP/PCL conduit, consisting of the innermost CeONP/PCL mixed layer, the outermost collagen layer, and the middle PCL layer [136]. They determined that CeONPs built a virtually low ROS microenvironment to trigger the ideal new vessel formation in long-range nerve defects in vivo. The presence of CeONPs enhanced the angiogenic status as confirmed by CD31 and CD34, markers of angiogenesis. Additionally, CeONPs induced neovascularization, as shown by the microvessel density, vessel-like structure, and density. Nevertheless, autografts were associated with the best angiogenesis in comparison with conduits. This observation could be due to the greater VEGF secretion from autologous nerves [136].

Despite these supportive studies forming the context of tissue engineering, there have some reports contradicting the angiogenic effects of CeONPs in the environment of non-cancer and cancer cells. In this regard, the study by Wang et al. can be observed [137]. They developed oligochitosan-coated cerium oxide nanoparticles (OCeONPs), which were loaded inside alginate injectable hydrogels as antioxidative agents, with an attempt to manage age-related macular degeneration. The in vitro release of the laden OCeONPs was in a controlled fashion over 60 days. The OCeONP-loaded hydrogels revealed strong antioxidative features and declined apoptosis in H_2_O_2_-treated ARPE-19 cells. In addition, these hydrogels could induce the suppression of the lipopolysaccharide-induced inflammation response in ARPE-19 cells. Notably, OCeONP-loaded hydrogels caused the inhibition of VEGF expression, as a pro-angiogenic factor, in human ARPE-19 and HUVECs [137]. In order to explain the anti-angiogenic potential of CeONPs, the existing literature sheds light on the concentration of CeONPs for their anti-angiogenic activity toward endothelial cells. Lord et al., for instance, documented that CeONPs alone or heparin-CeONPs at 50 μg/mL significantly inhibited growth by 10% or 20%–25%, respectively [138]. Dowding et al. employed water-based and hexamethylenetetramine-based methods to synthesize five different CeONPs. The proliferation of HUVECs did not rely on concentration but rather only upon exposure to CeONPs with the highest Ce^3+^/Ce^4+^. However, for the other four samples, those with round shapes significantly reduced the proliferation at 8.6 μg/mL, and the polygonal morphology displayed similar behavior at 8.6 μg/mL [139]. In the case of a cancer cell environment, where a great density of blood vessels is critical for the growth of the tumor, the role of pH is essential. For example, Giri et al. developed an in vivo ovarian cancer model and revealed a significant reduction of the vascular density after CeONP treatment relative to the control [140]. In addition, CeONPs were observed to decline microvascular density in an in vivo malignant melanoma model [141]. Such a dual property of CeONPs can be liked to their response to pH. In other words, CeONPs caused the formation of H_2_O_2_ at an acidic pH, while, on the contrary, the scavenging of H_2_O_2_ took place in a physiological pH. The accumulation of H_2_O_2_ is more likely to prevent the development of blood vessels [142].

### 4.3. Wound Healing and Skin Regeneration

Wound healing is a complex event in which oxidative stress causes delays. It has been noted that oxygen in tiny amounts accounts for the excessive ROS generation, leading to injured cells and tissues [35]. Several in vitro and in vivo studies exist concerning the suitability of CeONPs for the repair and regeneration of skin wounds via the inhibition of ROS accumulation [130,143,144]. In this setting, we can point out the work by Davan et al. [144], who reported that CeONPs of 160 nm with a spherical shape enhanced the wound quality (i.e., collagen deposition and wound closure rate) and appearance (lack of scar) by increasing the wound’s tensile strength in a rat model of skin wounds. Additionally, treatment with CeONPs resulted in the excellent nature and quality of the collagen in the wound area [144]. Further investigations have designed wound healing dressings with CeONPs. As a successful porous wound dressing, Naseri-Nosar et al. combined CeONPs with poly (ε-caprolactone)/gelatin films [145]. Their findings indicated that the film containing 1.5% CeONPs was considered the optimum dressing, as evidenced by the highest proliferation of L929 cells. Importantly, this construct displayed the desirable properties of wettability, tensile strength, water vapor transmission, and water uptake capacity. In Wistar rats, a two-week treatment of the wounds with 1.5% CeONP-containing dressing accomplished a remarkable closure to nearly 100% versus the sterile gauze with almost 63% wound closure [145]. The most recently published work about wound dressings has come from Sener et al.’s group, which fabricated a biomaterial system of zwitterionic cryogels (gels polymerized below freezing temperature conditions) loaded with CeONP- microRNA-146a (miR146a) for a better delivery method [146]. In order to avoid tough and brittle cryogels, chemical crosslinkers were removed, and, instead, the hydrogen bonding and electrostatic attractions between pendant groups of the zwitterionic polymers were used. In fact, these cryogels consisted of 3-((2-[methacryloyloxy]ethyl) dimethylammonio) propionate (CBMA) or (2-(methacryloloxy)ethyl)dimethyl-(3-sulfopropyl) ammoniumhydroxide (SBMA) and 2-Hydroxyethyl methacrylate (HEMA). The resultant cryogels seemed very flexible and biocompatible, with a self-healing ability and injectability, while keeping their sustained release of CeONP-miR146a, which depended on the monomer type and ratio. Zwitterionic cryogel could successfully deliver CeONP-miR146a topically, as evidenced by the elevated miR146a gene expression, reduced expression of pro-inflammatory cytokines IL6 and CXCL2, and promoted structural type 1 collagen. More importantly, the accelerated wound healing was achieved in a diabetic mouse model. The diabetic mouse skin after treatment demonstrated significantly increased modulus and overall strength, meaning that the healed wounds were of less rigidity and were not sensitive to future injury [146].

**Figure 3:**
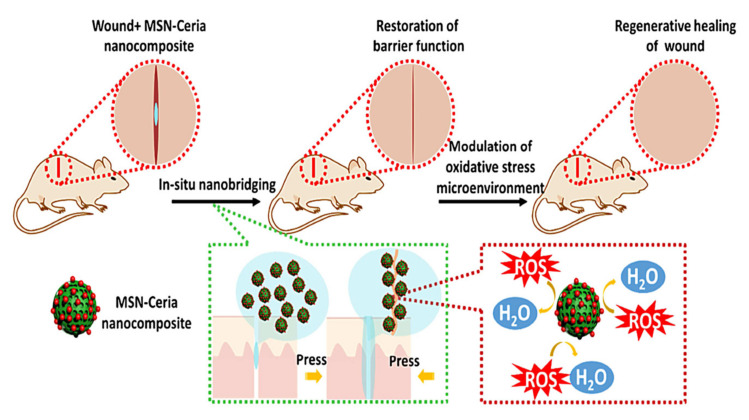
The contribution of mesoporous silica nanoparticles (MSN)-ceria to cutaneous wound healing and tissue repair. This tissue adhesive with an ROS-scavenging ability is designed by the regulated assembly of ultrasmall ceria nanocrystals on the surface of the MSNs. The initial stage starts when the edges of the wound are brought and kept together. Therefore, the recovery of the tissue barrier function accelerates. Then, MSN-ceria attenuates oxidative stress at the site of the injury and prepares a friendly microenvironment for tissue regeneration. In these events, three factors play a lead role: firstly, nano-sized assembly facilitates the development of functional hybrid materials [147]; secondly, MSN shows a remarkable tissue adhesive capacity, which is required for rapid wound closure [148,149]; thirdly, the immobilization of CeO_2_ nanocrystals prevents ROS from worsening with deleterious effects and potentiates the process of wound healing [150]. In this design, nanobridging for the recovery of barrier function is synergized with ROS-scavenging effects for control of the oxidative stress microenvironment, ultimately resulting in the substantial morphogenesis of skin appendages and the restricted formation of scar tissues. Reprinted with permission from [143].

With a view toward leveraging the intrinsic regenerative capabilities of the host for active wound healing, Wu et al. designed tissue adhesive using the assembly of ultrasmall ceria nanocrystals onto the surface of mesoporous silica nanoparticles (MSN) [143]. In this tissue-engineered product, MSN was responsible for rapid wound closure. Not only did the ceria nanocrystal-decorated MSN (MSN-CeONPs) display substantial tissue adhesion strength, but it could also significantly impede the exacerbation of ROS-mediated adverse effects, which, in turn, led to the efficient acceleration of the wound healing process. MSN-CeONPs revealed nanobridging effects and great CAT-mimetic activities. The ROS-scavenging capability of MSN-CeONPs was recoverable and protected cells from senescence. What is more, in the wound area, surprising regenerative healing was observed, with notable skin appendage morphogenesis and limited scar formation (Figure 3). In vivo evidence in rats indicated significantly low ROS signals and remarkably reduced local inflammatory responses, while a smooth appearance and improved quality of the healed skin were achieved upon treatment with MSN-CeONPs [143].

In another study, the wound nanobridge was explored by applying CeONPs with hollow and porous shells (termed as _Ah_CeONPs) [151]. This novel product originated from the hierarchical stimulation of the wound healing process, including the hemostasis, inflammation, and proliferation stages. In this scenario, the rough surfaces of _Ah_CeONPs played the role of a nanobridge to quickly close the wounds at the hemostasis stage. The hollow structure of the _Ah_CeONPs allowed for the multireflection of light inside the particles, the considerable enhancement of the light harvest efficiency, and the fundamental elevation of the electron-hole pair abundance. At the same time, the porous shells of the _Ah_CeONPs paved the way for electron-hole separation, ROS generation, the inhibition of wound infection, and the promotion of wound healing during the inflammation stage. The enzyme mimicking properties of the _Ah_CeONPs had the ability to diminish oxidative injury in the wound. _Ah_CeONPs contained loadings of _L_-arginine to give access to the nitric oxide source. The released _L_-arginine underwent conversion into nitric oxide under the catalysis of inducible nitric oxide synthase; the last two events improved the proliferation stage. Thus, _Ah_CeONPs with hollow structures, porous shells, rough surfaces, and high loading capacities can be wound nanobridges to stop bleeding, bond the wound’s edges, prevent wound infection under sunlight irradiation, and render epithelial cell proliferation in wound healing [151].

### 4.4. Controlled/Localized Delivery Systems

The design of biomaterial-based strategies, such as carriers for drug delivery systems, is essential in order to enhance regenerative medicine and tissue engineering. In fact, the regulation of biological activities in different cell types by means of bio-functional agents necessitates more research into drug delivery systems whereby active molecules are delivered to the target cells [152,153]. More specifically, these biomaterials can be loaded with genetic materials, and, at present, gene delivery is a new promising technology built on the concepts of tissue engineering to repair the impaired tissue or organ and developed by the incorporation of modified genes into the biocompatible three-dimensional matrices that can be further implanted or injected to initiate healing or regeneration processes. Current approaches have vastly focused on the transfection of target cells present in the local environment of impaired tissues by the use of both viral and nonviral delivery vehicles to drive the formation of new tissue. Despite this, as the viral mode causes a number of serious immunostimulatory consequences, such as patient safety risks, recent research attempts have been made for the application of the nonviral mode [98,154].

The majority of research studies concerning the delivery activities of CeONPs has been carried out in cancer cell settings. The use of CeONPs as carriers for delivering drugs or genes is an emerging strategy in tissue engineering applications and can provide a platform for tissue repair and function restoration. The work by Das et al. in 2016, for instance, was a pioneer in employing CeONPs for gene delivery [155]. They created dimethyldioctadecylammonium bromide (DODAB)—CeONP hybrids (CeO_2_/DODAB) as efficient nonviral gene delivery vectors for the transfection of plasmid DNA (pEGFPN1) in a number of cell lines, such as HEK293, MCF-7, and HepG2. The experimental data showed an average diameter of 461 nm. Additionally, the overall vector performance or transfection index of CeO_2_/DODAB was similar to lipofectamine 2000 and DOTAP (1,2-dioleoyl-3-trimethylammonium-propane) and superior to the calcium phosphate and DEAE-dextran utilized for transfecting small plasmids. The better gene delivery efficiency of this vector was highlighted by the promoted cellular uptake of the nanovector/DNA complexes through both clathrin- and caveolae-mediated endocytosis, as well as their subsequent release from the endosomes. In the case of the in vivo gene transfection efficiency and biocompatibility of the vectors, a mouse model was used for injecting the plasmid/nanovector complexes into the posterior tibialis muscles. In comparison with naked DNA injection, a 3.5-times higher fluorescence intensity was observed with CeO_2_/DODAB; however, the transfection efficiency was close to 17%, below the commercial in vivo-jeiPEI reagents. Therefore, the tranfection of genes could be accomplished by CeO_2_/DODAB nanovectors in vivo, and this complex holds promise for gene therapy approaches to tissue regeneration [155]. In the most recently published research, Zgheib et al. developed a nonviral miRNA delivery strategy based on CeONPs for wound healing and skin tissue engineering [156]. The cargo was the anti-inflammatory miR146a, and CeONPs played an antioxidant role. The animal studies showed that treatment with CeONP-miR146a accelerated the healing of diabetic wounds. Additionally, it had a lowering effect on inflammation and gave rise to increased angiogenesis. It is worth noting that the healing process observed in vivo did not compromise wound strength and elasticity [156]. As for drug delivery, Ma et al. have recently loaded _L_-arginine into the hollow spaces of _h_CeONPs since _L_-arginine can act as a substrate of a nitric oxide source that captures ROS and mediates proliferation throughout wound healing [151]. The loading capacity was 203.93 μg of _L_-arginine per μg of _h_CeONPs. The in vitro experiments confirmed the efficient production of nitric oxide by _h_CeONPs in a wound mimicking microenvironment. Furthermore, _h_CeONPs could effectively increase cell proliferation due to their dual role of antioxidant and nitric oxide generator [151].

### 4.5. Cerium Oxide as Advanced Theranostic Tool

The application of theranostics in medicine gives the opportunity of non-invasive imaging accompanied by simultaneous therapeutic intervention to achieve better clinical outcomes. There are a few studies focusing on the contribution of CeONPs to this emerging field [157]. CeONPs can exhibit SOD mimetic activity, whereby numerous diseases, namely stent restenosis and genetic mutations related to cancer, may be treated by inactivating the excess of ROS [158]. To this aim, Wu et al. developed novel Fe_3_O_4_ (core)/CeO_2_ (shell) theranostic NPs that could react with ROS and be recognized by magnetic resonance imaging (MRI). The diagnostic capability was provided by iron oxide (IO, MRI agent), while the therapeutic functionality was acquired by cerium oxide (anti-ROS capability) in one dose. This combination was also beneficial for tracking cerium oxide delivery to the disease site and assessing its biodistribution. What is more, these iron oxide-cerium oxide core-shell NPs (IO@CeO) acted as excellent contrast agents for MRI and had a good Ce^3+^ toCe^4+^ ratio. In addition to their considerable anti-ROS ability, they showed appropriate cell uptake and low cytotoxicity. These theranostic nanomaterials hold promise for the treatment and diagnosis of ROS-related inflammatory diseases including atherosclerosis, cardiovascular disease, rheumatoid arthritis, allergies, and other autoimmune diseases. The authors suggested conjugating antibodies or binding peptides to target inflammatory markers, such as vascular cell adhesion molecule 1 (VCAM-1) for atherosclerosis and neutrophil cytosolic factor 1 for rheumatoid arthritis [159]. Another study dealt with magnetite-CeO_2_ nanoconjugates based on NPs of IO interconnected with cerium oxide conjugates [158]. These nanoconjugates (average size of 8 nm) were synthesized in several stages, in which the NP coating with polyethyleneimine and its chemical activation and reticulation with glutaraldehyde were of utmost importance. Nanoconjugates were confirmed to benefit from superparamagnetic properties, and the incorporation of diamagnetic components into the system affected the saturation magnetization, which was still suitable for biomedical applications. The in vitro free radical scavenging activities of CeONPs increased upon the coating of NPs with PEI and conjugation with magnetite NPs. Animal investigations of mice shed light on the improved antioxidant activity in all organs and fluids, implying that the nanoconjugates were capable of alleviating oxidative stress. The magnetic properties along with free radical scavenging abilities make these nanoconjugates very interesting candidates for theranostic nanomedicine [158].

### 4.6. Regenerative Potential of Cerium Oxide-Based Nanozymes

**Figure 4:**
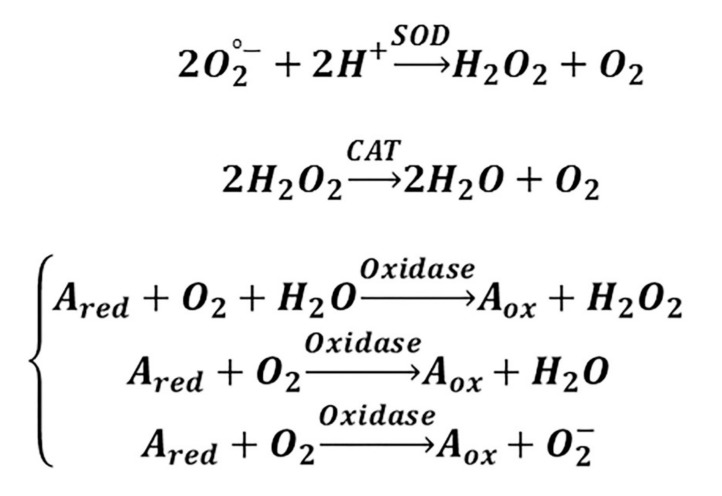
The SOD-, CAT-, and oxidase mimetic activities of CeONPs are found to be due to the co-existence of 3+ and 4+ oxidation states (chemical forms of Ce (III) and Ce (IV), respectively), which ultimately result in a redox couple in control of their antioxidant effect. This ability of Ce to switch between the 3+ and 4+ valence states is similar to the mechanism of redox enzymes, which make use of metals as co-factors in order to catalyze reversible redox reactions. The reactions that consist of redox cycles between 3+ and 4+ oxidation states provide this possibility for CeONPs to take part in catalytic reactions with O_2_^•−^, H_2_O_2_, and O_2_, thus showing the redox state-dependent mimetic activity of three major antioxidant enzymes [173].

Since the 1990s, scholars have investigated how to mimic the functionality and structural characteristics of biological enzymes [160]. So far, metal complexes [161], polymers [162], supramolecular systems [163], and bio-molecules [164] have been reported. In this context, inorganic NPs with enzyme-like properties, known as “nanozymes,” have attracted much attention. As shown in Figure 4, CeONPs have the ability to mimic SOD-, CAT-, and oxidase-like activity [165,166,167]. In reduction reactions, SOD catalyzes O_2_^•−^ into H_2_O_2_, which may undergo catalysis by CAT into H_2_O. Oxidase reaction refers to oxidizing a substrate by molecular oxygen, transformed to water or hydrogen peroxide [168]. Given the vital role of H_2_O_2_ in the regulation of proliferation and cell death [169,170,171,172], the multifunctional nanozyme activity of CeONPs is becoming the area of focus for many researchers in the field of tissue engineering. The characteristics of the studies which have applied CeONP-based nanozymes are summarized in Table 2.

**Figure 5:**
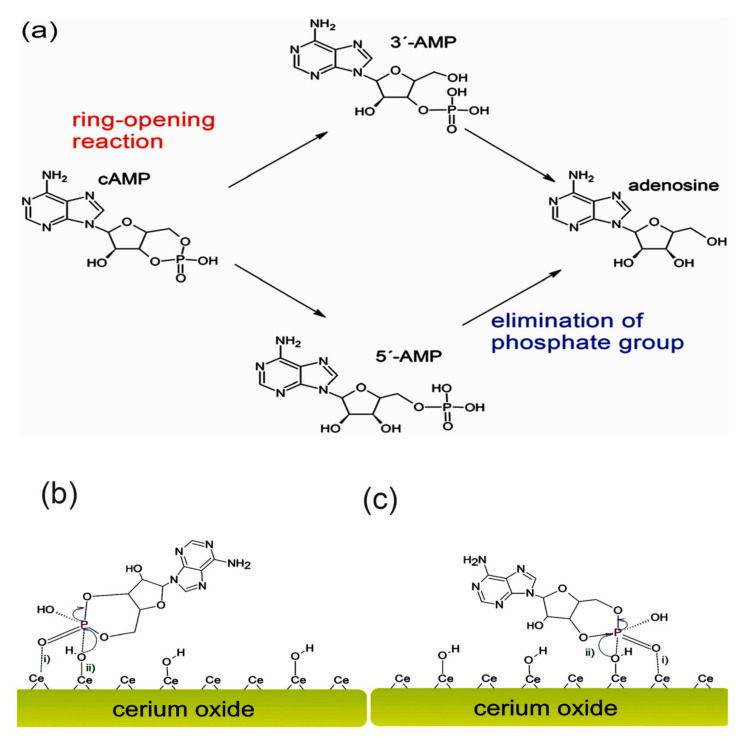
The transition states of the ring-opening reaction upon exposure to CeO_2_. The ring-opening reaction (**a**) is composed of two major mechanisms: the cleavage of the specific P–O bond via a transition state with a cyclic structure (i), as well as the nucleophilic attack on the P atom (ii) [199]. During the first interaction, the coordination reaction occurs between the phosphate group and a metal cation. This mechanism can explain the decontamination of toxic phosphotriesters [200]. With slight modifications, this model also shows the reaction resulting in 5′-AMP (**b**) and 3′-AMP (**c**). The excellent redox activities of the cerium cations in addition to the flexible structure of CeO_2_ can justify why CeO_2_ has considerably greater dephosphorylation activity than the oxides of neighboring elements. Reprinted with permission from [198].

DNA has been found as a useful material that can add functionality to bulk materials. Primarily, DNA is cleaved in three ways: hydrolysis, photochemistry, and oxidative reactions [180]. This characteristic provides a wide variety of approaches toward the degradation of DNA-based materials or the protection of these materials from insults. The remodeling of tissues during regeneration and repair involves intracellular events that culminate in DNA cleavage [181]. Accordingly, DNA modification including cleavage constitutes the central part of many applications, including tissue engineering [182], gene editing [183,184], biosensors [185,186], and therapeutics [187,188]. There are nucleases used for DNA cleavage at positions inside or outside its structure, respectively called endo- or exo-nucleases. In this regard, artificial nuclease mimics have provided the possibility of solving the disadvantages of protein-based nucleases (high cost and poor stability) and exploiting both oxidative and hydrolytic DNA cleavage [189]. These novel materials are founded on a variety of metal complexes [190,191], DNAzymes [192], and nanomaterials [193]. Cerium is commonly used for the hydrolytic cleavage of DNA, and CeONPs are multifaceted nanozymes [194,195]. Existing studies show the use of CeO_2_ for the dephosphorylation of simple organophosphates and energetically rich biomolecules, namely adenosine triphosphate [196,197]. In the recent study by Janoš et al. concerning the enzyme-mimicking activities of CeONPs, it has been highlighted that CeONPs are useful for the cleavage of more-resistant phosphoester bonds [198]. The authors investigated bonds present in 3′,5′-cyclic adenosine monophosphate (cAMP) since, among others, it efficiently mimics the phosphodiester bonds in nucleic acids. When cAMP was exposed to low-temperature-synthetized CeO_2_, rapid dephosphorylation of cAMP occurred to form adenosine as the final product (Figure 5). This dephosphorylation activity is exclusive to CeO_2_ and has not been determined for the oxides of neighboring lanthanides (i.e., La_2_O_3_, Pr_6_O_11_, and Nd_2_O_3_) or other metals. This type of CeONP was capable of destroying toxic compounds, like organophosphate pesticides (i.e., parathion methyl and paraoxon methyl), or dangerous chemical warfare nerve agents (i.e., soman and venomous agent X (VX)). Therefore, CeO_2_ as a phosphatase-mimicking nanozyme does not vary significantly between different phosphoesters. The nanocrystalline form of CeO_2_ definitely supports its enzyme-mimicking activity. Nevertheless, phosphatase-mimicking activity can also be found in classic CeO_2_ [198].

Furthermore, CeONPs have the ability to strongly adsorb single-stranded DNA [201,202] and nucleotides [203]. With this in mind, Xu et al. proved that smaller CeONPs (around 5 nm) could offer DNase I-like activity for the cleavage of single-stranded DNA oligonucleotides due to the presence of more defect sites, which might serve as active sites [204]. This potential of CeONPs as multiple turnover nanozymes was found to arise from the DNA-length dependent adsorption/desorption. Indeed, the mechanism of action includes DNA adsorption, cleavage, and subsequent desorption when the DNA length becomes less than 5-mer. Such evidence opens a new horizon for the nanozyme properties of CeONPs, from redox reactions to significant hydrolytic reactions [204].

## 5. Toxicokinetics of Cerium Oxide Nanoparticles

The seemingly simple concept of the scaffold refers to a structure that mechanically supports a construct during the building stages and then is removed at the end of a process, while not a part of this construction process. From the perspective of tissue engineering, biomaterials go beyond this, and a tissue-engineered scaffold is regarded as a biomaterial-based structure of defined size, chemistry, and architecture that creates a functional niche for the target tissues or organs [205]. One of the most important specifications of this scaffold is non-toxicity, which is determined similarly to the physicochemical properties of the scaffold. The ideal tissue engineered product must cause no toxicity and show minimal antigenicity in order to decrease the risk of rejection [206]. Notably, the most recent investigations on biomaterials deal with the question of how to modulate the immune response so as to control or prevent immunological reactions ending up with tissue or organ rejection [207]. In the literature, there is a body of evidence for and against the toxicity of CeONPs (Table 3). It is widely accepted that CeONPs show a poor toxicity profile, and, though they undergo cellular internalization, CeONPs do not activate inflammation or pose a risk of cytotoxicity [208,209,210]. Nevertheless, CeONP-related cell death has existed in previous reports [102]. In this section, the contribution of physicochemical parameters to the control of toxicity is discussed with more elaboration.

### 5.1. Effects of Size and Shape

The extant literature highlights the fact that sizes as well as shapes mainly account for the toxicity of NPs since changes in these factors are associated with varying degrees of cellular uptake and toxicity. In fact, smaller CeONPs show the potential to exert greater toxicity by virtue of their higher ratios of surface-to-volume, larger amounts of Ce^3+^, and faster kinetics to reach greater levels of cellular uptake. Lee et al. found that oleic acid-coated CeONPs of 3.8 nm with 44% Ce^3+^ quenched more H_2_O_2_ than those of 8.2 nm with 30% Ce^3+^ and attenuated H_2_O_2_-induced oxidative stress in human dermal fibroblast cells to a higher extent [219]. Selecting neuroblastoma cells as a cellular model, Kumari et al. evaluated the cytotoxicity of CeO_2_ particles at various sizes and showed that CeO_2_ particles within a nano range of 25 nm led to greater toxicity than those within a micro range of 3 μm [220]. The differential genomic effects of CeONPs with a particle size of 8 nm (M) and 58 nm (L) were investigated by Thai et al., whose study demonstrated that, relative to the M-particles, stronger antioxidant activities were obtained by the L-particles [221]. This observation may arise from a greater Ce^3+^ amount and a larger surface area to weight ratio for the L-particles. Notably, the M-particles carried the Warburg effect on human liver hepatocellular carcinoma HepG2, whereas none occurred in any L-CNPs. In comparison to the L-particles, more changes were caused by the M-particles in mitochondria dysfunction, apoptosis, the epithelial adherence junction, acute phase response, actin nucleation by ARP-WASP complex, the TCA cycle, and fatty acid levels by metabolomics. Nevertheless, the L-particles showed more activity in the pathways of hepatic fibrosis/hepatic stellate cell activation and Nrf2-mediated stress response [221]. In contrast with these findings, Peng et al. reported that CeONPs between 3nm and 5 nm were more/less cytotoxic than those of 6.6 nm, chiefly because of the agglomeration observed in smaller NPs [222].

More to the point, the CeONPs of the same particle size may be associated with varying toxicity based on their synthesis routes, which leads to different agglomeration tendencies. For example, precipitation and hydrothermal methods were employed to prepare CeONPs of the same size (3–5 nm) that caused pulmonary toxicity. More analyses in rats determined that CeONPs obtained by the precipitation method were more likely to result in acute inflammation owing to a higher amount of small aggregates and a greater rate of deposition in the deep lungs. In a different mechanism, the synthesis of CeONPs via the hydrothermal method generated ROS, which, in turn, resulted in inflammation and cytotoxicity in the early stage, as well as lipid peroxidation and pro-inflammation in the later stage. Such a disparity in toxicity mechanisms can be explained by changes in the nature of the agglomeration. Actually, the hydrothermal method produced CeONPs of larger agglomerates as compared to the precipitation method [222]. To control the CeONPs’ agglomeration, the addition of coating materials, including polymers or surfactants, seems influential. With this in mind, Lee et al. recorded the improved stability of poly(acrylic acid)- or oleic acid-coated CeONPs, as manifested by their repeated use over several months, which originated from the preservation of antioxidant activity [219]. With these experiments, they shed light on the importance of coating thickness in such a way that a thinner coating of poly(acrylic acid)/oleic acid enabled the NPs of 3.8 nm to quench more H_2_O_2_ than those of 8.2 nm, with a thicker coating of polyethyleneimine or polymaleicanhydride-alt-1-octadecene [219].

In addition to the particle size, the shape can play a considerable role in cytotoxicity. CeONPs are synthesized in different shapes, like spheres, cubes, rods, wires, and octahedral shapes [223]. CeONPs with elongated structures demonstrate a high aspect ratio that culminates in various chemical, electrical, magnetic, and optical features. As a result of these, these particles have different interactions with systems, such as cells and molecules, via distinct mechanisms [47]. The popularity of one-dimensional CeO_2_ nanostructures, such as NRs, nanowires (NWs), and nanotubes, is on the rise due to their marked redox and catalytic properties. The best instance is the study by Wang et al., who tested the toxicities of CeO_2_ nanocubes (NCs), nano-octahedra (NO), and NRs against HepG2 cells [224]. They came to the conclusion that NCs, NO, and NRs, in order from high to low, exerted toxicity in an inverse relationship to their specific surface area. What is more, the CeONPs with a smaller specific surface area could trigger more apoptosis, augment mitochondria’s membrane potential, augment ROS and GSH, and decline hydroxyl radical scavenging abilities [224]. A similar investigation into RAW264.7 cells was carried out by Forest et al. They found that only NRs, as opposed to NO and NCs/NO, showed toxicity dose-dependently in terms of lactose dehydrogenase release and tumor necrosis factor-alpha production, even at identical surface areas [225]. Intriguingly, Mahapatra et al. showed the contribution of shape to oxidative insult depending on their intracellular or extracellular actions [226]. They observed that CeO_2_ formed as nanospheres and NRs were able to scavenge more ROS than NWs since these particles could internalize to higher degrees within 24 h. Contrastingly, the suppression of ROS occurred extracellularly before the highly elongated NWs started cellular internalization, in order to protect human dental stem cells against ROS [226].

### 5.2. Effects of Surface Chemistry

Another important physiochemical parameter is the surface charge of CeONPs, which can take control of cell targeting, cell adhesion, cellular uptake, subcellular distribution, and toxicity. The surface charge of CeONPs can be arranged or altered to be positive, negative, or neutral by treatment with particular acidic/basic buffers or conjugation with polymers, biomolecules, or surfactants/stabilizers. The surface charge of CeONPs is measured as a zeta potential, which refers to the difference in the potentials between the dispersion medium and the stationary layer of fluid surrounding the dispersed particles [227]. Overall, the literature defines a zeta potential greater than 30 mV or less than −30mV for electric stabilization. However, a stable dispersion of NPs can also be obtained at low zeta potentials, especially when high molecular weight polymers, surfactants, or stabilizers are used (known as steric stabilization). Hence, a minimum zeta potential of ±20 mV is desired for electrostatic and steric stabilization [228].

In general, the cellular uptake of NPs comes about as the result of two main steps: binding to the cell membrane and, ultimately, internalization. The first step largely depends on the surface charge of both CeONPs and cell membranes, so that CeONPs with great surface charges are tightly bound to the membrane via electrostatic interaction [229]. On the surfaces of cells, there are negatively charged sulfated proteoglycans with their core proteins, for the most part. These molecules become anchored in the membrane and form links to one or more anionic glycosaminoglycans. Accordingly, the positively charged NPs interact with the cellular surface to a great extent via electrostatic forces. It has been reported that CeONPs with negative charges and a zeta potential of −43 mV could show a larger cellular uptake than those with less negative or positive charges [230]. Despite the fact that the anionic cell membranes tend to exert repulsion to CNPs with negative charges, particular cationic sites on the cell surface form clusters and bind to these CNPs. This event, as a localized neutralization, can lead to the bending of the cell membrane and then endocytic uptake. As a confirmation, the preferential uptake of negatively charged CeONPs by tumor cells has been found in previous studies [230,231]. In another investigation, the superior uptake of transferrin-coated CeONPs with positive charges by the A549 human lung adenocarcinoma cell line was observed, relative to WI-38 normal human diploid fibroblasts [232]. It has been highlighted by Fröhlich that positively charged NPs undergo preferential internalization by non-phagocytic cells, whereas negatively charged NPs do so by phagocytic cells [229]. Therefore, the cell type is also responsible for the cellular uptake of CeONPs.

Apart from these, the interaction energy between CeONPs and the cell surface is of the utmost importance for cytotoxicity. Comparatively, Li et al. analyzed seven metal oxide NPs and determined this order of cytotoxicity from low to high: Al_2_O_3_ < TiO_2_ < CeO_2_ < ZnO < SiO_2_ < CuO < Fe_2_O_3_ NPs [233]. This suggests that the lower the interaction energy barrier with the cell surface, the higher the cytotoxicity. Such a relationship appears to be moderated by the NP’s adhesion to the cell surface so that easier adhesion results in greater cytotoxicity [233]. Following internalization, CeONPs localize in a specific part within a cell that relies on the CeONP’s surface charge. As an example, Asati et al. found both positively charged and neutral dextran-coated CeONPs to be internalized in healthy (H9C2 and HEK293) and cancer cells (A549 and MCF-7) [234]. The positive CeONPs affected the lysosome and cytoplasm, and they were toxic against all cells except MCF-7. The presence of the neutral CeONPs in the cytoplasm caused no marked cytotoxicity. The negatively charged CeONPs presented the strongest potential to localize in the lysosome and inhibit A549 growth for the most part [234].

It is worth paying attention to the monitoring of the CeONP’s surface charge since changes in pH, time, temperature, and CeONP concentration (for example, by the adsorption of OH^−^ ions on their surface) have been shown to make positive charges (kinetically stable state) shift to negative charges (thermodynamically stable state), thereby carrying various cytotoxic impacts. Vincent et al. assessed the surface charge reversal of CeONPs in response to the pH variations implemented by means of acidic and basic buffers [235]. Their findings revealed no change in zeta potential as CeONPs were exposed to an acidic buffer. When it came to an alkaline buffer, the positive charge switched to negative after the elevation of the pH. The isoelectric point corresponded to pH 10. They also considered time and determined that aging for 40 days caused the zeta potential of positively charged CeONPs to become negative. If this was extended to 220 days, the zeta potential of CeONPs dropped to within the range of highly negative (−26 to −36 mV), irrespective of their initial surface charge. To justify this pH- and time-dependent event, they pointed out the substitution of positively charged species in the CeONP’s surface (H^+^) with negatively charged counterions (OH^−^). A similar observation was made when the temperature increased. Indeed, the zeta potential of CeONPs decreased, and the surface charge underwent a shift at 65 °C. Additionally, the zeta potentials reversed from positive to negative values following the increase of the annealing temperature from 100 to 900 °C, which was indicative of a quicker rate of OH^−^ adsorption at annealing temperatures. In terms of the most common explanation, reducing the concentration from 1mM to 10 μM resulted in behavior similar to the zeta potential (shift to negative values). Note that varying the particle size to the micro range was followed by a negative zeta potential [235].

From a different perspective, whatever the surface charge is, culture with cells in a medium generates a negative surface charge in CeONPs because they may have a tendency to conjugate with protein or phosphate in the medium. This finding is reported by Das et al., whose study addressed the induction of angiogenesis by CeONPs [129]. Their observation was the alteration in zeta potentials of both positive (+44 mV) and negative (−20 mV) CeONPs to a new negative value (−9.23 mV) when administered to endothelial basal media-2 supplemented with 2% fetal bovine serum [129]. Concerning this effect, Naganuma and Traversa pinpointed the transition of both positive (rich in Ce^3+^) and negative (dominant Ce^4+^ regions) CeONPs covering poly-_L_-lactide acid scaffolds into negative upon the use of Eagle’s minimum essential medium containing 10% fetal bovine serum [109]. Thus, the binding of CeONPs to proteins can afford to completely remove the electrostatic difference between the positively and negatively charged CeONPs. However, in this case, they took the role of Ce^3+^/Ce^4+^ ratio into account more than the surface charge. This is because human osteoblast-like cells (MG63) proliferated on the CeONPs with high content levels of Ce^4+^, whereas the viability of hMSCs was in decline after the administration of high Ce^3+^ CeONPs. [109]. To delve into the idea of protein interaction, much more research has been considered for positively or negatively charged CeONPs. It has been demonstrated that the highly positive CeONP surface tends to adsorb larger amounts of protein (e.g., transferrin) which, in turn, brings about better cell adhesion through ligand-receptor-mediated interactions [232]. However, while positively charged CeONPs are more potent at adsorbing protein (bovine serum albumin), a higher cellular uptake (e.g., cell line A549) occurs only in cases of negatively charged CeONPs with low protein adsorption [230]. Again, Naganuma and Traversa observed with a different phenomenon where protein (bovine serum albumin) adsorption did not differ between positively and negatively charged CeONPs, and it did not contribute to the prevention of cell adhesion and growth [230]. Nevertheless, this may imply interactions with other proteins or biomolecules, since CeONPs acquire a new identity when they are exposed to biological environments consisting of numerous bioactive components, particularly proteins. Singh et al. showed the significance of a biologically important phosphate buffer, which could alter both particle size and zeta potential [236]. To provide more details, the elevation of the phosphate concentration from 10 to 100 μM was followed by an increase in the CeONP’s particle size, with a stable suspension (from 24 to 290 nm) and a decrease in the zeta potential. These events might stem from the electrostatic attraction of phosphate anions on the positively charged surfaces of CeONPs, which eventually culminated in charge neutralization [236].

### 5.3. Effects of Hidden Factors

There are some studies that relate the difference in cytotoxicity to other physicochemical parameters of CeONPs and cell types. It is more likely that CeONPs with various morphologies have varying crystal facets in connection to their stability and reactivity. Accordingly, Naganuma designed an experiment including nanopolyhedra, NC, and NR CeO_2_ fabricated with certain crystal planes and surface areas, such as (111)/(100) and 82.4 m^2^/g, (100), and 93.2 m^2^/g, besides (111)/(100) and 163.7 m^2^/g, respectively [237]. Nanopolyhedra containing a large amount of Ce^4+^ exhibited low levels of toxicity against human promyelocytic leukemia cells (HL60) and high levels of CAT mimetic activity. In contrast, both NCs and NRs rich in Ce^3+^ could afford to elevate SOD mimetic activity [237].

Some others pay attention to the role of the optimal length and aspect ratio of high aspect ratio-NPs in cytotoxicity. Ji et al. synthesized CeO_2_ NRs and NWs and found no toxicity for the NRs with low aspect ratios when cultured with human myeloid monocytic cells (THP-1) [47]. In the case of the NRs with intermediate aspect ratios, there was only IL-1β production, without any cell death. Surprisingly, two samples of NWs with high aspect ratios induced notably greater cell death versus shorter NRs. In their study, the critical length and aspect ratio, which resulted in lysosomal damage, were 200 nm and 22, respectively. The leading cause behind this event was the formation of stacking bundles for narrow NRs/NWs as a result of the Van der Waals force and dipole–dipole attractions of parallel-aligned NRs/NWs [47].

Apart from these, the sharp edges of CeONPs can afford to impair cells mechanically. This effect is exemplified by high aspect ratio CeONRs, which could initiate progressive pro-inflammation and toxicity against THP-1 cells, as characterized by lysosomal damage along with IL-1β production [47]. However, Dowding et al. presented contrary evidence when they treated HUVEC cells with hexamethylenetetramine (HMT)-coated CeONPs with polygonal shapes and sharp edges. These very particular morphologies failed to affect cell availability significantly as compared to HMT-CeONPs with round shapes [139].

## 6. Conclusions

Over the last few decades, nanotechnology has produced remarkable advantageous outcomes, particularly in the interesting field of regenerative medicine, which, in turn, allows for the synthesis of the next generation of nanostructured biomaterials. Notably, nanocomposite hybrid scaffolds, fabricated by the incorporation of NPs into biocompatible tissue engineered constructs, have drawn the attention of scholars across the globe. The reasonable inclusion of CeONPs in the present therapeutic strategies for regenerative medicine and tissue engineering will be associated with an unprecedented advancement for tissue repair. Some examples of the actual possibilities are discussed in this review for bone, heart, skin, eyes, vessels, the brain, and the nervous system, in which CeONPs with new and regenerative oxidant or antioxidant properties open up a wide range of application opportunities. The distinctive redox ability of CeONPs defines many promising biological activities and biomedical applications. The strongest features of CeONPs are in fact their intrinsic antioxidant and oxidant capacities, which exert a large number of constructive effects, enabling CeONPs to truly have an important role compared with other existing options. In tissue engineering and regenerative medicine, we would like to shed light on three main aspects: (i) some evidence is present that CeONPs are able to carry out tissue regenerative activity due to their oxidant potential; (ii) CeONPs can be utilized for the healing of different impairments caused by reactive species; (iii) CeONPs hold promise for tissue engineering since these materials act as initiators to trigger signaling pathways in the actions of stem cell differentiation and angiogenesis. However, what matters most in tissue engineering is the manufacturing of vascularized and innervated tissues, which has not been well addressed as yet. To this end, both synthesis and fabrication techniques should be integrated into the hierarchical stimulation of angiogenesis and differentiation processes, as shown previously for the use of _Ah_CeONPs in wound healing [151].

However, several toxicity issues remain that should be addressed for the forthcoming clinical practice. In this regard, both physiochemical properties and the biological environment are responsible for the CeONP’s variable cytotoxicity. As for the size and shape of CeONPs, the small size is directly correlated to the large surface to volume ratio, which provides an excellent platform for cells to interact. Additionally, there is plenty of Ce^3+^ on the surfaces of CeONPs with a small size that can afford to scavenge both cellular and intracellular ROS [100]. Conversely, some observe the toxic effect of CeONPs with the highest value of Ce^3+^, which not only increases ROS generation but also interferes with their attachment to cells [238]. Moreover, studies exist in support of the CeONP’s capability to cause toxicity in cellular models, with no mention of size and morphology [239]. Instead, other parameters, such as aggregation, aspect ratio, surface charge, entry rate, cell culture environment, cell type, and storage condition, are taken into account [226,235]. Therefore, there is no solid trend on how CeONPs are involved in toxicokinetic interactions which eventually lead to toxicity. On the basis of this review, a comprehensive characterization of synthesized CeONPs under storage conditions and in response to influential variables, including medium pH, protein, and concentration, should be conducted during toxicological tests. Since biosystems often consist of complex interactions between biomolecules, particularly proteins, it is of the utmost importance to make CeONP–protein interactions clear and develop bioinspired CeONPs for avoiding interactions with plasma proteins.

Apart from toxicity itself, the lack of standardized assays lies behind the complexity and discrepancy of the CeONP’s toxicological behavior and poses many challenges for introducing CeONP-containing tissue engineering implants into widespread clinical applications. As noted earlier, the toxicity of CeONPs depends to a large extent on the administered dose. Hence, they are usually applied below their threshold concentrations, where harmful effects occur. Nevertheless, the uptake and accumulation of CeONPs inside cells and tissues are reported in many studies. If this event persists over an extended period of time, CeONPs can reach their threshold concentrations, which leads to detrimental consequences for cells, tissues, and organs. From a different point of view, to the best of our knowledge, no world-wide standards exist for nano-specific health risk assessments, and manufacturers are dedicated to evaluating the health hazards associated with their NP-based products. Hitherto, the measurement procedures are self-supervised and not nano-specific. Legislative changes concerning chemicals are required to harmonize the notification of product information by manufacturers, produce data requirements for classification criteria, labels, and other forms of warnings, and develop safety data sheets. Thus, there is a vital need for an increased knowledge level, in vivo research design, and precautionary measures for CeONP-based applications, especially when chronic bioaccumulation may be involved.

## Figures and Tables

**Table 1:** Cerium oxide (CeO_2_) nanoparticles (CeONPs) in different tissue engineering formulations targeting differentiation of stem cells.

Formulation	Role of CeONPs	Cell Type	Target Tissue	Tissue Repair Process	Remarks	Ref.
CeONP-incorporated hydroxyapatite (HA) coatings	Additive to scaffold	Bone marrow stromal cells (BMSCs)	Bone	Constructive remodeling	Enhancement of cell viability Attenuation of cell apoptosis Concentration-dependent improvement of osteogenic differentiation Restoration of gene and protein expression downregulated by H_2_O_2_ Inhibition of osteoclastogenesis Recovery of SOD activity Reduction of ROS generation Suppression of malondialdehyde formation	[108]
Cancellous bone containing poly-L-lactic acid and CeONPs	Additive to scaffold	Mesenchymal stem cells (MSCs)	Bone	Constructive remodeling	Improvement of cell proliferation Inhibition of cell apoptosis Activation of the calcium channel Increased stability of HIF-1α	[70]
Poly-L-lactide scaffold functionalized by CeONP layers	Additive to scaffold	Human mesenchymal stem cells (hMSCs)	Bone	Constructive remodeling	Surface charge and hydrophobicity/hydrophilicity defined the cell behaviors and cell–biomaterial interactions.	[109]
CeONP-incorporated HA coatings	Additive to scaffold	Bone marrow-derived mesenchymal stem cells (BMMSCs) & RAW264.7 macrophages	Bone	Constructive remodeling & fibrous capsule formation	Better osteogenic behaviors of BMMSCs Promotion of osteogenic differentiation Tendency toward an M2 phenotype Downregulation of ROS production Reduction of inflammatory reactions	[110]
CeONPs coated onto Ti-6Al-4V substrates	Additive to scaffold	Bone MSCs & RAW264.7 macrophages	Bone	Constructive remodeling & fibrous capsule formation	Enhanced osteogenic behavior of bone MSCs Positive contribution of higher Ce4+ concentration to cell behavior Promotion of osteogenic differentiation Increased anti-inflammatory cytokines Suppression of proinflammatory cytokines Reduced ROS production Upregulation of osteoconductive molecules in macrophages	[111]
CeONPs	Dispersion in medium	human adipose derived-mesenchymal stem cells (hAd-MSCs)	Skin	Constructive remodeling	Enhanced tensile strength of the acellular dermal matrices Impregnation of the matrices with CeONPs Increased growth and survival of hAd-MSCs Improvement of free radical scavenging High amount of collagen	[112]
CeONPs & Samarium (Sm)-doped CeONPs	Dispersion in medium	Neural progenitor cells	Nerves	Constructive remodeling	Cellular up-take of CeONPs and Sm-doped CeONPs Temporary cytoprotection of CeONPs against a pro-oxidant Inhibition of neuronal differentiation Interference with cytoskeletal organization Developmental neurotoxicity hazard	[113]
Citrate-stabilized CeONPs	Dispersion in medium	Primary mouse embryonic fibroblasts	-	Constructive remodeling	Enhanced proliferative activity of primary cells Reduction of intracellular ROS during the lag phase of cell growth Modulation of major antioxidant enzymes	[114]
CeONPs	Dispersion in medium	BMSCs	Bone & adipose	Constructive remodeling	Time- and dose-dependent increase in the viability of BMSCs Time- and dose-dependent inhibition of osteogenic differentiation and adipogenic differentiation	[115]
CeONPs	Dispersion in medium	Cardiac progenitor cells (CPCs)	Heart	Constructive remodeling	No alteration of the cellular growth and differentiation Protection of cells against oxidative insults	[116]

**Table 2:** Summary of studies on the effectiveness of CeONPs during oxidative damage by mimicking three key antioxidant enzymes in various tissues.

Formulation	Target Tissue	Type of Study	Natural Enzyme	Remarks	**Ref.**
CeONP powders	Arteriole	In vivo	CAT SOD	Increasing microvascular reactivity Decreasing microvascular oxidative stress in a high ROS environment	[174]
CeO_2_-doped bioactive glasses	Bone	In vitro	CAT	Enhancing the degradation of H_2_O_2_ for glasses containing more CeO_2_ Reconverting some Ce^4+^ to Ce^3+^ in hydrogen peroxide	[175]
CeO_2_-doped bioactive glasses	Bone	In vitro	CAT	Preventing the interconversion process between Ce^3+^ and Ce^4+^ by phosphate groups Lower CAT mimetic activity in the presence of phosphate groups	[176]
CeO_2_-incorporated hydroxyapatite coatings	Bone	In vitro	SOD	Eliminating H_2_O_2_-evoked intracellular ROS generation Downregulating lipid peroxidation content of BMSCs Alleviated SOD activity in BMSCs by the incorporation of CeO_2_ in the hydroxyapatite coating	[108]
CeONPs	Brain	In vivo	CAT SOD Oxidase	Displaying different enzyme-mimetic activities Great neuroprotection Association of NP surface coatings with different enzyme-mimetic activities and biological effects Potent antioxidant activity of NPs stabilized with equal ratios of citric acid/ethylenediamine triacetic acid (EDTA)	[177]
CeONPs	Heart	In vitro	SOD CAT	Apparent silence of the internalized CeONPs inside CPCs Acting as a defense upon oxidative insults Self-regenerating antioxidant mechanism for CeONPs, involving redox cycles between the Ce^3+^ and Ce^4+^ oxidation states	[116]
Flame-made ceria NPs and ceria/bioglass hybrid NPs	Skin	In vitro	CAT SOD	Linking CO-oxidation activity to surface defects and Ce^3+^ sites Regulating the enzymatic activity of the NPs by the Ce^3+^ content Association of the active species regeneration with the cycling of the ceria oxidation states Tailoring the antimicrobial properties of CeONPs by controlling the cerium oxidation state	[178]
CeONP functionalized PCL-gelatin nanofiber mesh	Skin	In vitro	SOD	Altered SOD activity Dissolution of uncross-linked gelatin Showing the antioxidant effect in different buffer systems	[179]
Ceria nanocrystals decorated MSN	Skin	In vitro	SOD CAT	Highly efficient dose-dependent ROS-scavenging activity Great CAT-mimetic activity Good dispersion state Mutual reinforcement between SOD- and CAT-mimetic activities Recoverable ROS-scavenging capability	[143]

**Table 3:** Pharmacokinetic studies during in vitro or in vivo toxicity.

Formulation	Size (nm)	Zeta Potential (mV)	Design	Test	Model	NP Concentration	Time	Signs of Toxicity	Ref.
CeONPs	6, 12, 1000	-	In vitro	3-(4,5-Dimethylthiazol-2-yl)-2,5-diphenyltetrazolium bromide (MTT) assay	HT22 hippocampal nerve cell line	0.0002–20 μg/mL	5–240 min	Protection of cells against oxidative stress No relationship with particle size Maximal protection after about 10 min	[34]
CeONPs	15, 25, 30, 45	-	In vitro	MTT assay	BEAS-2B human lung epithelial cell, T98G human glioblastoma multiforme cell, embryonic cardiomyocyte cell line H9C2	5, 10, 20, 40 μg/mL	24, 48, 72, 96 h	Decline of cell viability in a time- and dose-dependent manner No relationship with particle size and cell type	[49]
CeONPs	7, 14, 94	-	In vitro	Trypan blue exclusion dye analysis	Human monocyte cell line U937	5, 200 μg/mL	24, 72, 144 h	Reducing cell proliferation within 24 h No cell impairment up to 144 h No relationship with particle size and concentrations	[100]
CeONPs	100–200	−19, −61	In vitro	MTT assay	Prostate cancer cell line PC-3, L929 murine fibroblast cell line	0.001–5 μg/mL	24, 72 h	More cytotoxicity of hydrothermal NPs (Ce^4+^) to the normal and prostate cancer cells than hydrolysis NPs (Ce^3+^)	[211]
Custom-synthesized CeONPs	2.9	−23.5	In vivo	Fluorescence-activated cell sorting, microvasculature staining, and visualization	Mice	10, 30 mg/kg	1 day before and 0, 3, and 7 days after disease induction	No evidence of liver pathology Lack of CeONP’s effect on immune cell distributions No uptake by endothelial cells	[212]
Spherical and rod-shaped CeONPs	7–9.5	11.7 to 40.6 in distilled water, −10.4 to −15.3 in Dulbecco’s Modified Eagle’s Medium (DMEM), −19.72 to −32.55 in Holtfreter’s medium	In vitro	Measurement of IL-1β activity	Human myeloid cell line THP-1, bone marrow-derived macrophages	100 μg/mL	24 h	As for aspect ratio ≥22, lysosomal damage and progressive effects on IL-1β production and cytotoxicity in THP-1 cells	[213]
			In vivo	Acute and sub-chronic toxicity (measurement of lipopolysaccharide-induced CXC chemokine, IL-1β, and TGF-β1 levels)	Zebrafish larvae, mouse	10, 20, 40, 80 μg particles per 50 μL suspensions	40 h, 21 days, 44 days	Mice: acute lung inflammation for CeO_2_ nanospheres and NRs Significant IL-1β and TGF-β1 production for the highest aspect ratio No pulmonary fibrosis Zebrafish larvae: growth inhibition, decreased body weight, and delayed vertebral calcification for highest aspect ratio	
Ceria nanocrystals decorated mesoporous silica NPs	<5 nm	~2	In vitro	3-(4,5-dimethylthiazol-2-yl)-5-(3-carboxymethoxyphenyl)- 2-(4-sulfophenyl)-2H-tetrazolium (MTS) cell proliferation assay, Immunofluorescence analysis for cell morphology and interactions	EA.hy926 endothelial cells or HaCaT human keratinocytes	0, 25, 50, 100 μg/mL		Being highly biocompatible Cells with a well-organized cytoskeleton, and cell–cell and cell–surface interactions Negligible cytotoxicity to cells	[143]
			In vivo	Immunofluorescence staining for CD68	Rats	10 mg/mL	days	Decreased local inflammatory response at the wound site Decreased oxidative stress	
CeONPs	3–5, 15–20, 30, 50–20 in Phosphate-buffered saline (PBS) & 18, 194, 370, 192 in culture medium	10.8, −21.9, 3.95, −5.11 in PBS & −6.08, −10.86, −9.98, −5.9 in culture medium	In vitro	MTT assay	CCL30 (squamous cell carcinoma) cells	250 μM	60 min	Being non-toxic to the CCL-30 cell line	[214]
CeONPs	-	-	In vivo	RT^2^ profiler PCR arrays, measurement of cytokine levels	Rats	0.1, 0.3, 1.0, 3.0 mg/m^3^	28, 90 days	No marked genotoxicity and apoptosis based on gene expression Inflammatory responses in liver and kidney tissues	[215]
CeONPs	-	-	In vivo	Systemic toxicity	Rats	250, 500, 1000 mg per implantation site	28 days	Minimal local tissue reactions No systemic toxicity or in vivo micronucleus induction in bone marrow Migration from the implant sites and deposition in liver, lungs, spleen, and kidneys	[216]
CeONPs	-	-	In vivo	Acute toxicity	Rats	50, 100, 200, 400 mg/kg	14 days	No significant changes in the activity of liver enzymes, hepatic and renal histopathology, and hematological parameters	[217]
CeONPs	5−6	46.9	In vitro	MTT assay, confocal microscopy, flow-cytometry	Human glioma (U87MG), breast cancer cell lines (BT 474 and SK BR 3)	0–200 μg/mL	24, 48 h	No significant cell mortality Noteworthy differences between the images of U87 MG, SK BR 3, and BT 474 cells Specific changes of cell granularity, cell sizes, and metabolic signaling Relationship between cell granularity and concentration Main NP uptake by U87MG	[218]
			In vivo		Quail embryos chorioallantoic membrane (CAM)	20, 100, 400 μg/mL	24 h	No malformation of the CAM vasculature Significant effect on the ectoderm Concentration-dependent hemorrhage High biocompatibility	

## Abbreviations

cAMP	3′,5′-cyclic adenosine monophosphate
BMSCs	Bone marrow stromal cells
CDs	Carbon dots
CAT	Catalase
CeONPs	Cerium oxide nanoparticles
CeONRs	Cerium oxide nanorods
CPCs	Cardiac progenitor cells
DNA	Deoxyribonucleic acid
DODAB	Dimethyldioctadecylammonium bromide
GSH	Glutathione
HA	Hydroxyapatite
hAd-MSCs	Human adipose derived mesenchymal stem cells
hMSCs	Human mesenchymal stem cells
HIF-1α	Hypoxia inducible factor
HUVECs	Human umbilical vein endothelial cells
IL	Interleukin
IO	Iron oxide
MRI	Magnetic resonance imaging
MSN	Mesoporous silica nanoparticles
miR	MicroRNA
mRNA	Messenger ribonucleic acids
NCs	Nanocube
NO	Nano-octahedra
NPs	Nanoparticles
NRs	Nanorods
NWs	Nanowire
NSCLC	Non-small-cell lung cancer
Nrf2	Nuclear factor erythroid 2-related factor 2
PCL	Polycaprolactone
PEI	Polyethylenimine
PLGA	Poly lactic-co-glycolic acid
Ref-1/APE1	Reduction-oxidation factor 1-apurinic/apyrimidinic endonuclease
ROS	Reactive oxygen species
Sm	Samarium
SFSNPs	Silk fibroin nanoparticles containing sulforaphane
SOD	Superoxide dismutase
TGF-β	Transforming growth factor
VEGF	Vascular endothelial growth factor
